# Associations between shift work patterns and sleep disturbance: an analysis of cross-sectional data from UK Biobank

**DOI:** 10.1136/bmjopen-2025-102976

**Published:** 2026-01-21

**Authors:** Xianqi Li, David W Ray, Simon D Kyle, Karl Smith-Byrne, Leah Holmes, Annie Keane, Mahboubeh Parsaeian, Ruth C Travis, Rebecca Richmond

**Affiliations:** 1 Cancer Epidemiology Unit, Nuffield Department of Population Health, University of Oxford, Oxford, UK; 2 Oxford Centre for Diabetes, Endocrinology and Metabolism, Radcliffe Department of Medicine, University of Oxford, Oxford, UK; 3 National Institute for Health and Care Research Oxford Biomedical Research Centre, John Radcliffe Hospital, Oxford, UK; 4 Sir Jules Thorn Sleep and Circadian Neuroscience Institute, University of Oxford, Oxford, UK; 5 Vocal, Manchester University NHS Foundation Trust, Manchester, UK; 6 MRC Integrative Epidemiology Unit, Bristol Medical School, University of Bristol, Bristol, UK; 7 Population Health Sciences, Bristol Medical School, University of Bristol, Bristol, UK

**Keywords:** EPIDEMIOLOGY, SLEEP MEDICINE, OCCUPATIONAL & INDUSTRIAL MEDICINE, Cross-Sectional Studies, PUBLIC HEALTH, Insomnia

## Abstract

**Abstract:**

**Objective:**

To investigate associations between shift work patterns and sleep disturbance, and to assess if the association is modified by demographic factors, socioeconomic factors, anthropometric and lifestyle factors, health conditions or sleep traits.

**Design:**

Analysis of cross-sectional data obtained from the UK Biobank baseline assessment.

**Setting:**

UK Biobank, a large-scale prospective cohort study which recruited half a million participants aged 40–69 years between 2006 and 2010 from across the UK.

**Participants:**

A total of 285 175 employed or self-employed participants at baseline (2006–2010), including 148 296 (52.0%) females and 136 879 (48.0%) males. The sample comprised 94.0% White, 0.7% Mixed race, 0.36% East Asian, 2.0% South Asian, 1.8% Black and 0.89% from other ethnic backgrounds.

**Outcome measures:**

Sleep disturbance was defined as the presence of both insomnia and excessive sleepiness symptoms.

**Results:**

A total of 42 181 (14.8%) participants had sleep disturbance defined based on insomnia and excessive sleepiness. 236 200 (82.8%) were non-shift workers, while 48 975 (17.2%) were shift workers, which included 24 062 (49.1%) working day shifts only, 17 940 (36.6%) working night shifts sometimes or usually, and 6973 (14.2%) working night shifts always. Compared with non-shift workers, all shift workers had higher multivariable-adjusted odds of sleep disturbance: (non-night shifts: OR in model 3 (OR) 1.21 (95% CI 1.16 to 1.27); sometimes/usually night shifts: OR 1.37 (95% CI 1.30 to 1.44) and always night shifts: OR 1.50 (95% CI 1.38 to 1.63)). The association between shift work pattern and sleep disturbance was modified by age (p_interaction_<0.0001), ethnicity (p_interaction_=0.0005) and smoking status (p_interaction_=0.04).

**Conclusions:**

Shift work is associated with a higher odds of sleep disturbance compared with non-shift work in all participants, with greatest odds observed among those always working night shifts. The association was stronger among individuals who were younger than 55 years old, from an ethnic minority background and never smokers. Future large-scale longitudinal studies are needed to further investigate these associations.

**STRENGTHS AND LIMITATIONS OF THIS STUDY**

- The large sample size enabled detailed assessment of associations between shift work patterns and sleep disturbance across sociodemographic groups.
- The cross-sectional design limits the ability to infer temporal or causal relationships.
- Sleep disturbance was defined using self-reported measures of insomnia and excessive daytime sleepiness, rather than full International Classification of Sleep Disorders-3 criteria.

## Introduction

 Shift work, which involves varying work hours across morning, evening and night shifts, can vary in its intensity and speed of rotation. According to the sixth European Working Conditions Survey, 21% of employees in Europe reported working shifts, with 19% working nights at least once a month.^1^ More recently, data from the UK Office for National Statistics in 2022 revealed that approximately 27% of the workforce, or around 8.7 million people, worked evening or night shifts, with 4% working exclusively during these hours.^2^

Shift work sleep disorder (SWSD)^3^ is a chronic condition that affects individuals who work outside of traditional daytime hours, including night shifts, rotating shifts and irregular work schedules.^4^ A systematic review and meta-analysis with 29 studies from 16 countries worldwide, including both low- and middle-income and high-income settings, reported that the overall prevalence of SWSD was 26.5%.^5^ The disorder is primarily characterised by insomnia, excessive daytime sleepiness and difficulties in maintaining a regular sleep-wake cycle, all of which are aggravated by the misalignment between the body’s internal circadian rhythm and the demands of shift-based work.^6^ As global reliance on 24-hour operations grows, particularly in industries such as healthcare, transportation and manufacturing, SWSD has emerged as a significant concern for both individuals and organisations alike.^7 8^ In addition, UK Biobank studies have reported that, compared with non-shift workers, shift workers have an 11% higher risk of incident cardiovascular disease,^9^ a 22% higher risk of depression^10^ and a 16% higher risk of anxiety.^10^ Usual night shift workers have 44% higher risk of diabetes,^11^ a 21% higher risk of myocardial infarction,^12^ a 23% higher risk of asthma^13^ and 29% higher odds of non-alcoholic fatty liver disease.^14^

Individual vulnerability to sleep disturbance in shift workers could vary based on sociodemographic factors and other sleep and circadian traits, for example, chronotype and shift work patterns. Some smaller studies with limited sample sizes and occupation types have reported higher risks of sleep disturbance among shift workers and/or night shift workers, but few have comprehensively examined those factors which influence susceptibility to shift work-associated sleep disorder.^15^,^18^ For instance, a Taiwanese cross-sectional study with 164 400 employees aged 25–65 years old suggested that, compared with a fixed day shift pattern, a fixed night shift pattern was associated with greater risks of sleep problems in both males and females.^18^ To the best of our knowledge, the relationship between specific shift work patterns and sleep disturbance in different sociodemographic groups remains unquantified in large, population-based studies.

There is cross-sectional evidence from a study of 238 shift workers suggesting that individuals with a late chronotype, whose physiological and behavioural rhythms naturally occur later in the 24-hour cycle, may experience less circadian misalignment and sleep disruption when working night shifts compared with those with an early chronotype.^19^ A similar association, where individuals with a later chronotype may adapt better to working night shifts, has been suggested in another cross-sectional study involving 388 nurses.^20^ Considering the potential importance of chronotype in developing personalised prevention strategies,^21^ it is necessary to replicate these initial findings in a larger study.

In this UK Biobank study of up to 285 175 individuals, we aim to investigate associations between shift work patterns and sleep disturbance. Additionally, we assessed whether the association was modified by demographic, socioeconomic, anthropometric and lifestyle factors, health conditions or sleep traits.

## Methods

### Study design and participants

UK Biobank is a prospective population-based cohort study that recruited more than 500 000 adults aged 40–69 years between 2006 and 2010. Invitations were sent to approximately 9 million people from the UK National Health Service (NHS) registers, and approximately 5.5% responded positively. Each participant attended one of 22 assessment centres across England, Scotland and Wales, where they completed a series of questionnaires on a computerised touchscreen interface, followed by a face-to-face interview conducted by trained health professionals to gather additional data.

During this baseline assessment, participants reported on their lifestyle, medical conditions, work hours and demographic information, and provided details on their medical history, health status and medication intake. The present analysis involved cross-sectional data from the baseline assessment.

### Main exposure assessment: shift work

During the baseline survey, participants were asked about their employment status (paid employment, self-employed or other). Those who were employed were further asked if their work involved shift work. Shift work was defined as a work schedule outside normal daytime working hours of 9:00–17:00, which may involve working in the afternoon, evening or night, or rotating through these kinds of shifts. Participants who indicated ‘sometimes’, ‘usually’ or ‘always’ were then asked whether this included night shifts. Night shifts were defined as a work schedule that involves working through the normal sleeping hours, for instance, working through the hours from 12:00 to 6:00. For both questions, response options were ‘never/rarely’, ‘sometimes’, ‘usually’ or ‘always’, with additional options for ‘prefer not to answer’ and ‘do not know’. The participants who selected ‘prefer not to answer’ or ‘do not know’ were excluded from further analysis. Based on those two questions, we categorised participants’ current shift work status as ‘non-shift workers (never or rarely shifts)’, ‘day shift workers (shift but never or rarely night shifts)’, ‘night shifts (sometimes/usually)’ and ‘night shifts (always)’.

### Outcome: sleep disturbance

SWSD is currently defined based on criteria outlined in the International Classification of Sleep Disorders (ICSD-3)^22^ and the Diagnostic and Statistical Manual of Mental Disorders, fifth edition (DSM-5).^23^ According to the (ICSD-3),^22^ SWSD is clinically assessed using three key questions: (a) Do you have a recurring work schedule that overlaps with your usual sleep time? (b) If yes, does this cause insomnia and/or excessive sleepiness due to a reduced amount of sleep? (c) If yes, has this persisted for at least 3 months? Participants are classified as having SWSD if they respond ‘yes’ to all three questions. The DSM-5^23^ defines SWSD more broadly as a pattern of sleep disturbance caused by unconventional work hours, leading to insomnia and/or excessive sleepiness.

To approximate this, in the UK Biobank we obtained information on insomnia symptoms and excessive daytime sleepiness. In the UK Biobank, insomnia symptoms were assessed by asking participants, ‘Do you have trouble falling asleep at night or do you wake up in the middle of the night?’ with response options of ‘never/rarely’, ‘sometimes’, ‘usually’ and ‘prefer not to answer’. Excessive sleepiness was assessed by asking, ‘How likely are you to doze off or fall asleep during the daytime when you don’t mean to (eg, when working, reading or driving)’ with responses of ‘never/rarely’, ‘sometimes’, ‘often’, ‘do not know’ and ‘prefer not to answer’. Participants were categorised as having insomnia or sleepiness symptoms if they answered ‘sometimes’ or ‘usually’/‘often’ to this question, and participants were excluded if they selected ‘do not know’ or ‘prefer not to answer’. In this study, we define sleep disturbance as the presence of both insomnia and excessive sleepiness symptoms to increase the specificity of outcome assessment.

### Covariates

We considered possible confounding factors, as suggested by literature review, which were collected via questionnaires or verbal interviews at baseline, including:

1. Sociodemographic factors: Age, sex, ethnic origin (White and individuals from ethnic minority backgrounds, including Mixed race, East Asian, South Asian, Black and others), education (above A levels, A levels, below A levels), neighbourhood-level socioeconomic status as measured by the Townsend index of deprivation, household income group (less than GBP18 000, GBP18 000–GBP30 999, GBP31 000–GBP51 999, GBP52 000–GBP100 000 and greater than GBP100 000) and marital status (living with partner or not).
2. Anthropometric and lifestyle factors: Body mass index (BMI; calculated as weight in kilograms divided by height in metres squared), smoking (never, former and current), alcohol intake (daily, 1–4 times a week, sometimes, never/special occasion), physical activity (low, moderate and high), working hours (more than 40 hours or not).
3. Medical conditions: self-reported overall health (poor, fair and excellent/good), self-reported hypertension and diabetes mellitus. The latter two were defined by asking if they had been told by a doctor that they had certain medical conditions.
4. Other sleep-related characteristics and chronotype: Participants self-reported sleep traits on a touch-screen questionnaire at baseline, which included sleep duration (short <7 hours, normal 7–9 hours and long >9 hours),^24^ getting up in the morning (levels of difficulties), morning or evening person (definite morning, intermediate morning, do not know (middle), intermediate evening, definite evening), nap during the day (never, sometimes, usually) and snoring (yes or no). For chronotype, responses of ‘do not know’ were included as a middle chronotype, while ‘prefer not to answer’ were set as missing. For all other variables, responses of ‘do not know’ and ‘prefer not to answer’ were set as missing. We include each of the sleep traits in the baseline characteristic description but only include sleep duration and chronotype as confounding factors in the regression analysis. The reasons are: (1) night shift workers may not wake up in the morning, (2) they are more likely to sleep during the day and (3) snoring could be a symptom or a consequence of a sleep disorder rather than a contributing factor.

### Statistical analysis

Baseline characteristics of the participants were described using the mean (SD) for continuous variables and proportions (from complete data) for categorical variables by current shift work schedule. Additionally, we reported baseline characteristics by demographic groups (age, sex and ethnicity) across different shift work types, outcome (presence or absence of sleep disorder) and chronotype (morning or evening). For questionnaire responses, ‘do not know’ and ‘prefer not to answer’ were treated as missing values, with the exception that ‘do not know’ responses for chronotype were coded as intermediate chronotype.

Multivariable logistic regression models were performed to estimate ORs and 95% CIs for the association between shift work status and sleep disturbance. We constructed several models to estimate ORs and their 95% CIs: Model 1 adjusted for age and sex; Model 2 adjusted for age, sex, ethnicity and education and Model 3 further adjusted for additional sociodemographic factors (Townsend deprivation index and marital status), anthropometric and lifestyle factors (BMI, smoker, alcohol, physical activity and working hours), medical conditions (overall health, hypertension and diabetes) and sleep traits (sleep duration and chronotype). All the models were conducted using complete data. The amount of missingness for each variable is highlighted in online supplemental table S1, and for each model in table 1. Multicollinearity of the variables included in the models was assessed using variance inflation factors (VIFs) derived from ordinary least squares regression including the same set of predictors.

**Table 1:** Current shift work and odds of sleep disruption in the UK Biobank (N=285 175)

	N	Non-shift workers (n=236 200)	Shift workers (n=48 975)		
			Day shift workers (n=24 062)	Nightshift (sometimes/ usually) (n=17 940)	Night shift (always) (n=6973)
Total cases*		33 012	4366	3530	1573
Model 1: age group- and sex-adjusted OR (95% CI)	285 175	1.00	1.39 (1.34 to 1.44)	1.61 (1.55 to 1.68)	1.90 (1.79 to 2.01)
Model 2:+ education- and ethnicity-adjusted OR (95% CI)	252 687	1.00	1.33 (1.28 to 1.38)	1.52 (1.45 to 1.58)	1.75 (1.65 to 1.88)
Model 3: multivariable-adjusted OR (95% CI)†	197 405	1.00	1.21 (1.16 to 1.27)	1.37 (1.30 to 1.44)	1.50 (1.38 to 1.63)

* The number of cases varies depending on the missing values of covariates in each model, as a complete-case analysis was used.

† Additionally adjusted for Townsend deprivation index, household income, marital status, BMI, smoking, alcohol drinking, physical activity levels, working hours, overall health, hypertension, diabetes, sleep duration and chronotype.

BMI, body mass index.

To assess whether each covariate in model 3 could modify the adjusted association between shift work pattern and sleep disorder, we used a log likelihood ratio test to compare models with and without cross-product interaction terms; corresponding p values were based on χ^2^ statistics. Stratified analyses were conducted accordingly, stratifying by each covariate in model 3. BMI (kg/m²) was categorised into three groups: <25, 25–30 and >30.

In the sensitivity analyses, we conducted regression analyses restricted to participants with the same complete data across all models. We also conducted adjusted regression analyses without the adjustment of sleep duration, due to the complex relationship between sleep duration and insomnia.

All data management and analyses were conducted with Stata V.18 (StataCorp). All hypothesis tests were two‐tailed with α=0.05. Online supplemental file 1 outlines the original study protocol and the completed Strengthening the Reporting of Observational Studies in Epidemiology Checklist for observational cross-sectional studies can be found in online supplemental file 2.

### Patient and public involvement

Our National Institute for Health and Care Research programme includes an NHS Shiftworker Research Advisory Group (PPIEP) led by Vocal, a not-for-profit health research engagement organisation. The PPIEP group supports our aim to quantify the association between shift work patterns and sleep disorders and to identify vulnerable populations. They provided insights into how NHS staff describe and manage shift work. Moving forward, they will assist our research team in applying the epidemiological evidence from this study to inform the design of interventions, which may help NHS shift workers better adapt to their shift work schedules. The Group will also work with us to co-develop engagement approaches and resources targeted for key audiences including shift workers and their families, employers and policymakers.

An occupational health specialist has been involved as an advisor from the beginning of the research.

## Results

**Figure 1:**
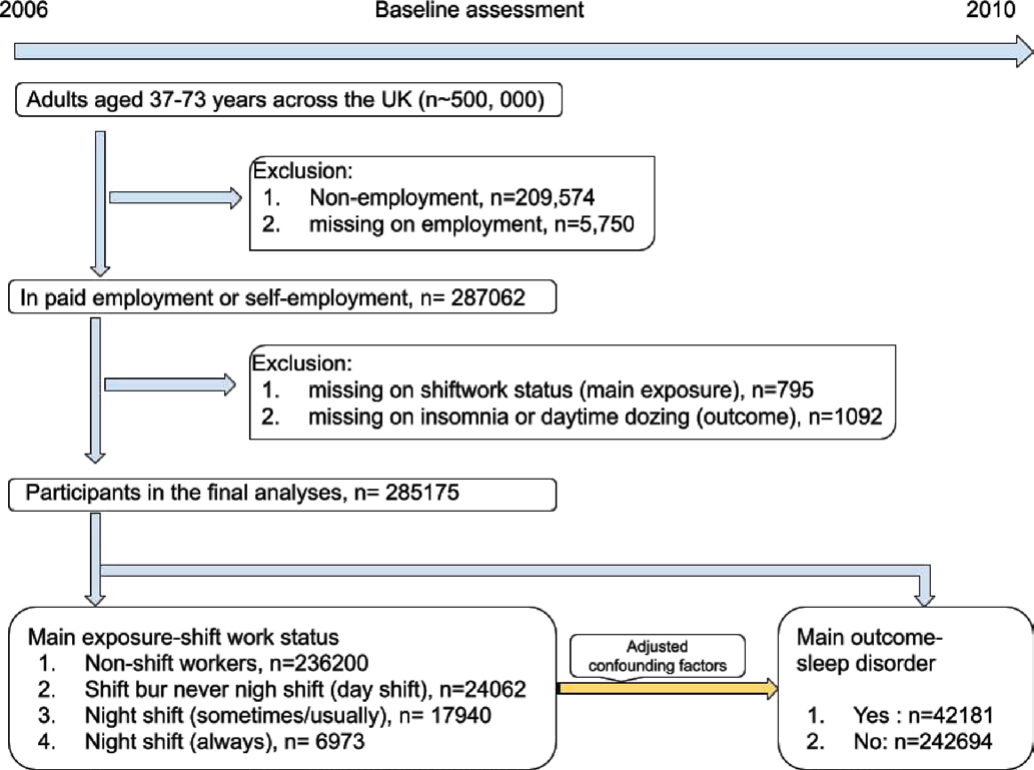
Summary of study design.

For the present study, we restricted our analyses to 287 062 UK Biobank participants who were in paid employment or self-employed at baseline. After excluding individuals with missing data on shift work type (n=795) and missing information on insomnia and daytime dozing (n=1092), 285 175 participants were included in the final analyses (figure 1). Online supplemental table S1 shows the baseline of the characteristics of this analytical sample, while online supplemental table S2 displays the characteristics by presence or absence of sleep disturbance.

We identified 42 181 (14.8%) prevalent cases of sleep disturbance among all employed participants in the UK Biobank. Of the total participants, 236 200 (82.8%) reported no engagement in shift work, while 48 975 (17.2%) were shift workers. Among the shift workers, 24 062 (49.1%) exclusively worked day shifts, 17 940 (36.6%) engaged in night shifts occasionally or usually and 6973 (14.2%) always worked night shifts.

Overall, shift workers tended to be younger, more likely to be male, to have a higher degree of material deprivation (as measured using the Townsend deprivation index), and less likely to live with a partner than those who did not do shift work (online supplemental table S1). Individuals who were from ethnic minority backgrounds and less well educated were engaged in more frequent and intense shift work. Shift workers also exhibited higher BMI, were more likely to be current smokers, engage in high levels of physical activity, work longer hours (>40 hours per week), report poorer overall health and have hypertension and diabetes. However, they were less likely to consume alcohol daily. Shift workers were more likely to have short sleep duration (<7 hours), nap during the day and experience snoring. Additionally, night shift workers reported greater difficulty getting up in the morning, and evening chronotypes were twice as common among night shift workers compared with non-shift workers.

Among non-shift workers, 33 012 (14.0%) were classified as having sleep disturbance, compared with 9469 (19.3%) among shift workers (table 1). Within the shift worker group, the prevalence of sleep disturbance was 18.1% in day shift workers, 19.7% in those who worked night shifts sometimes or usually, and 22.6% in those who always worked night shifts. Table 1 shows the odds of sleep disturbance by shift work status in all employed UK Biobank participants. Compared with non-shift workers, in the age group- and sex-adjusted model 1, there were higher odds of having sleep disturbance in day shift workers who never or rarely undertook night shifts (OR: 1.39, 95% CI 1.34 to 1.44), night shift workers (sometimes/usually) (OR: 1.61, 95% CI 1.55 to 1.68) and night shift workers (always) (OR: 1.90, 95% CI 1.79 to 2.01). The effect estimates were attenuated with additional covariate adjustment, but odds remained significantly elevated for all shift workers. In model 2, the odds were 1.33 (95% CI 1.28 to 1.38), 1.52 (95% CI 1.45 to 1.58) and 1.75 (95% CI 1.65 to 1.88), respectively. In model 3, the odds were 1.21 (95% CI 1.16 to 1.27), 1.37 (95% CI 1.30 to 1.44) and 1.50 (95% CI 1.38 to 1.63) for the three shift work types. There was limited evidence for multicollinearity among covariates included in the models, with a mean VIF value below 2 for the most adjusted model (model 3) (online supplemental table S3).

**Figure 2:**
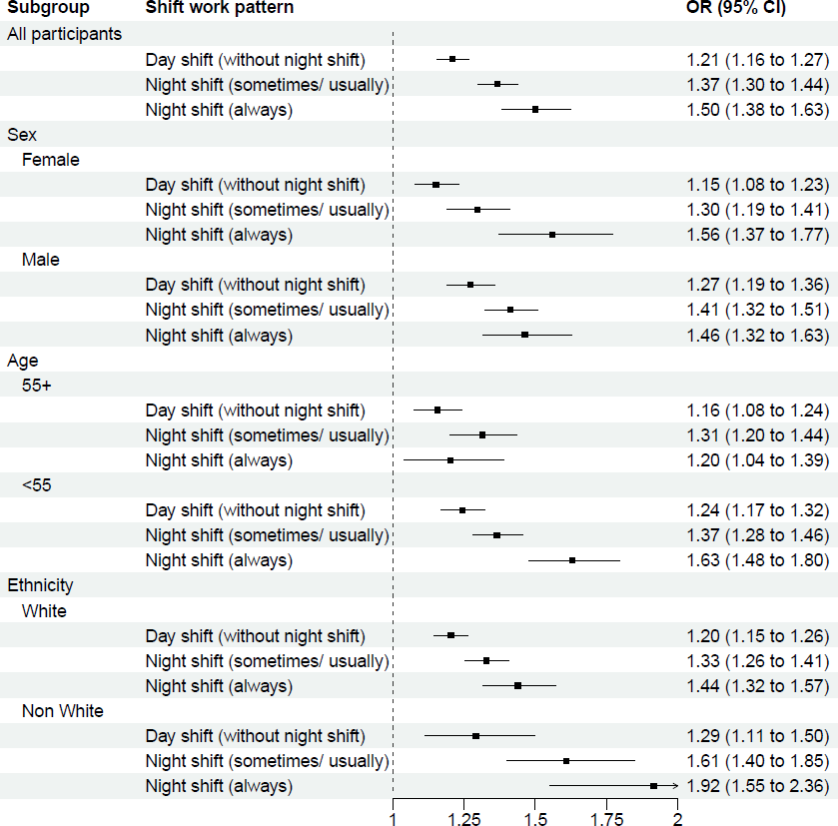
Multivariable-adjusted* associations between shift work types** and sleep disturbance, stratified by demographic subgroups: age, sex and ethnicity. *Logistic regression. Adjusted for age, sex, ethnicity, education, Townsend deprivation index, household income, marital status, BMI, smoking, alcohol drinking, physical activity levels, working hours, overall health, hypertension, diabetes, sleep duration and chronotype, except for the subgroup variable itself. **Reference group: Non-shift workers. BMI, body mass index.

Stratified analyses of the association between shift work and sleep disturbance are shown in figures2^6^ and online supplemental table S4–S23. This analysis revealed potential interactions by age (p_interaction_<0.0001, online supplemental table S5), ethnicity (p_interactio_=0.0005, online supplemental table S6) and smoking status (p_interaction_=0.0370, online supplemental table S13). Night shift work (always) was more strongly associated with sleep disturbance among those aged <55 years (OR 1.63; 95% CI 1.48 to 1.80) than among those aged >55 years (1.20 (95% CI 1.04 to 1.39)). Similarly, for night shift work (sometimes/usually), the association was stronger among those aged <55 years (1.37 (95% CI 1.28 to 1.46)) than among those aged >55 years (1.31 (95% CI 1.20 to 1.44)). For ethnicity, night shift work was more strongly associated with sleep disturbance among individuals from ethnic minority backgrounds, with higher odds of sleep disturbance for both always (1.92 (95% CI 1.55 to 2.36)) and sometimes/usually working night shifts (1.61 (95% CI 1.40 to 1.85)), compared with White participants (1.44 (95% CI 1.32 to 1.57) and 1.33 (95% CI 1.26 to 1.41), respectively). Further stratification (online supplemental table S7) showed that, within each shift work type compared with non-shift workers, except for East Asians, all ethnic minority subgroups (Mixed race, South Asian, Black and others) exhibited stronger association of sleep disturbance than White participants. For smoking status, the association between night shift work (always) and sleep disorder was weaker among current smokers (1.14 (95% CI 0.91 to 1.41)) than never smokers (1.66 (95% CI 1.49 to 1.85)). However, no significant interactions were observed across other demographic (sex, education, Townsend index of deprivation, marital status), lifestyle (BMI group, alcohol intake, physical activity, working hours), health-related factors (overall health condition, hypertension, diabetes) and sleep traits (sleep duration, chronotype) (p_interaction_ range: 0.0618–1.0000; online supplemental table S4–21).

**Figure 3:**
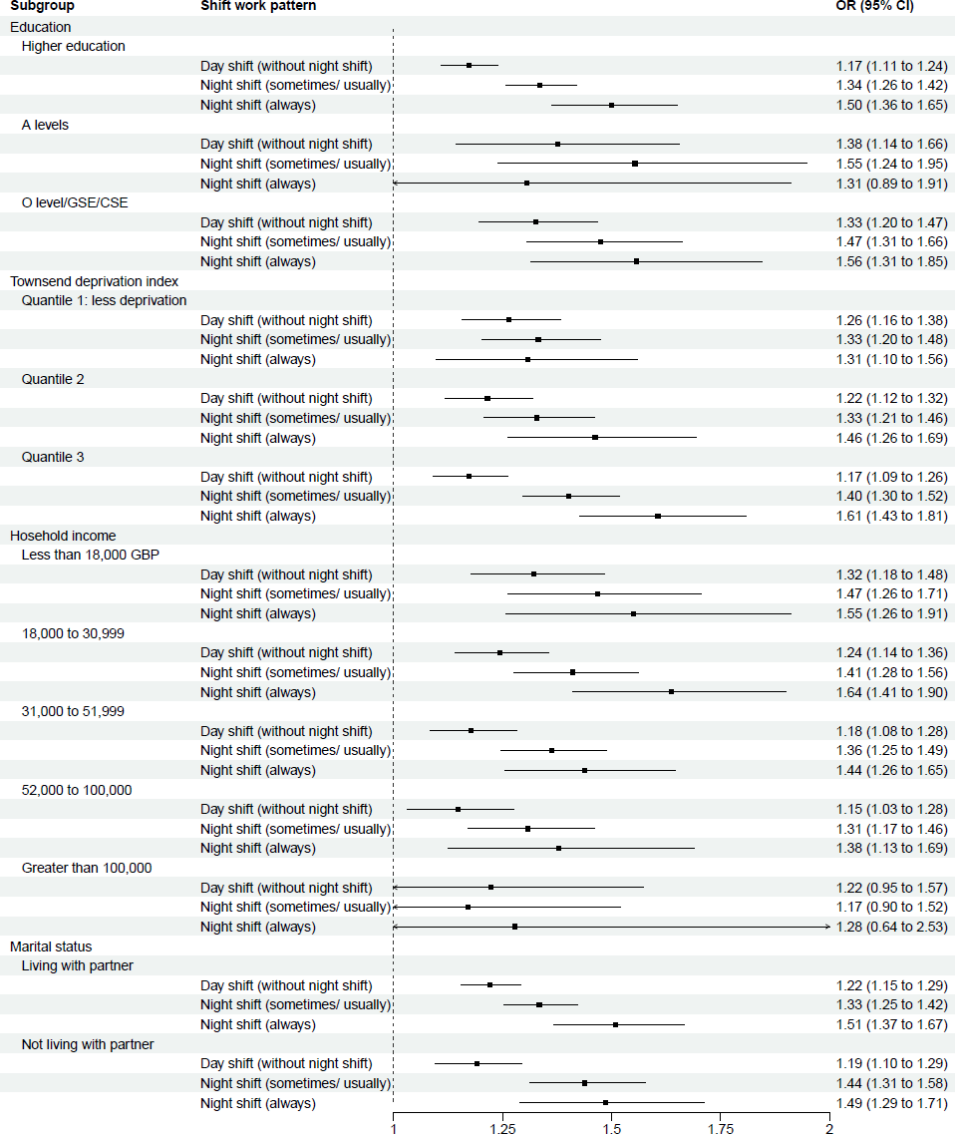
Multivariable-adjusted* associations between shift work types** and sleep disturbance, stratified by socioeconomic subgroups. *Logistic regression. Adjusted for age, sex, ethnicity, education, Townsend deprivation index, household income, marital status, BMI, smoking, alcohol drinking, physical activity levels, working hours, overall health, hypertension, diabetes, sleep duration and chronotype, except for the subgroup variable itself. **Reference group: Non-shift workers. BMI, body mass index. O-level/GSE/OSE, Ordinary level/General Certificate of Secondary Education/Certificate of Secondary Education (secondary school qualification); A-level, Advanced level (post-16 qualification).

**Figure 4:**
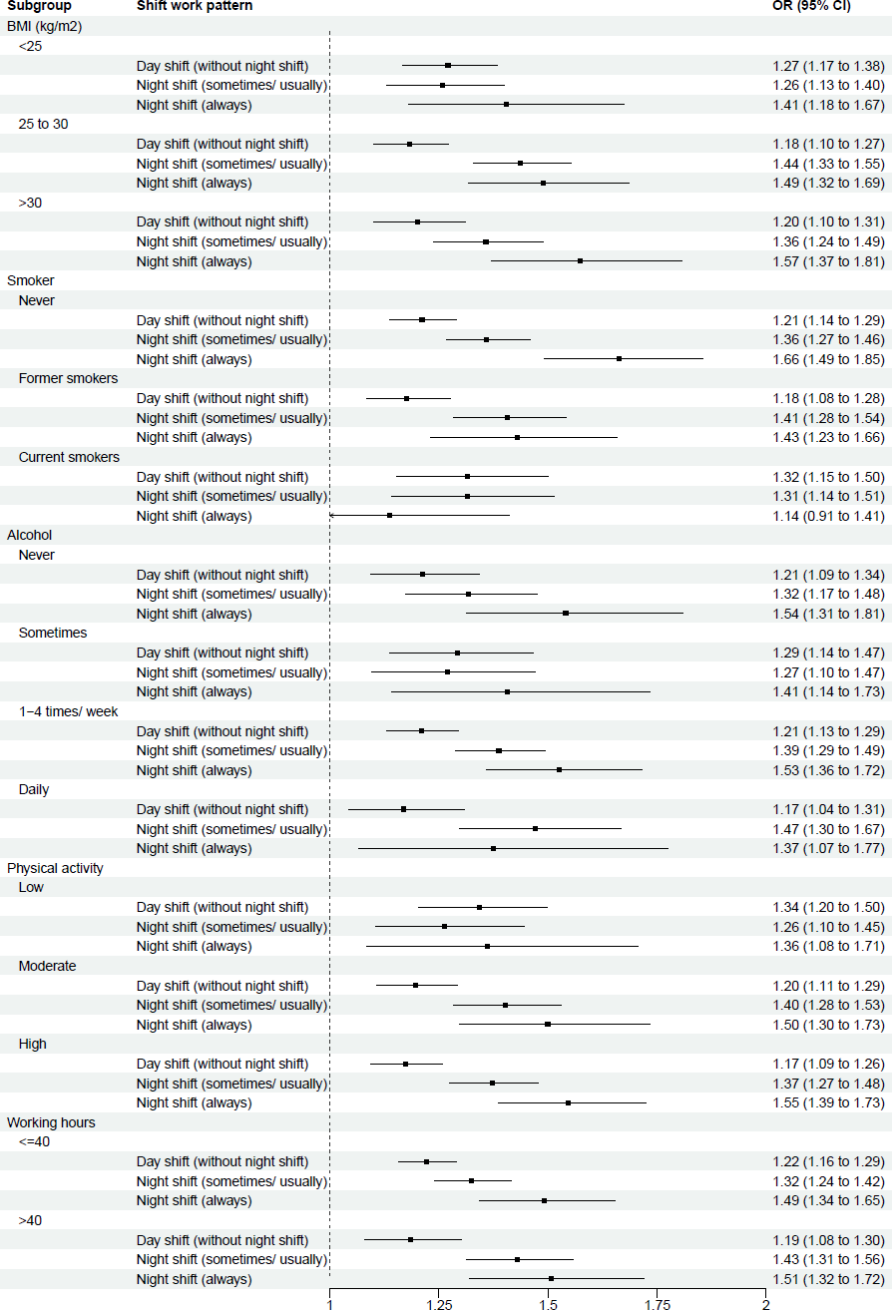
Multivariable-adjusted* associations between shift work types** and sleep disturbance, stratified by anthropometric and lifestyle subgroups. *Logistic regression. Adjusted for age, sex, ethnicity, education, Townsend deprivation index, household income, marital status, BMI, smoking, alcohol drinking, physical activity levels, working hours, overall health, hypertension, diabetes, sleep duration and chronotype, except for the subgroup variable itself. **Reference group: Non-shift workers. BMI, body mass index.

**Figure 5:**
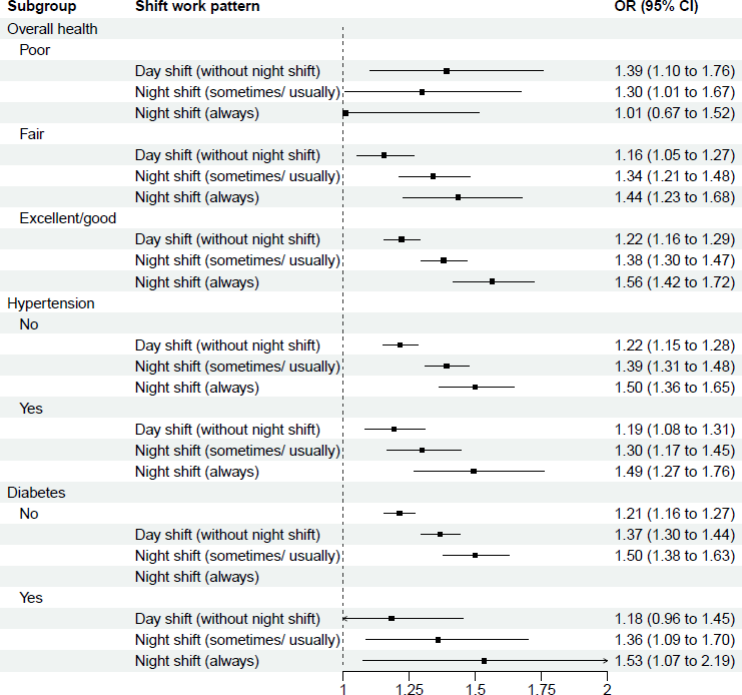
Multivariable-adjusted* associations between shift work types** and sleep disturbance, stratified by health condition subgroups. *Logistic regression. Adjusted for age, sex, ethnicity, education, Townsend deprivation index, household income, marital status, BMI, smoking, alcohol drinking, physical activity levels, working hours, overall health, hypertension, diabetes, sleep duration and chronotype, except for the subgroup variable itself. **Reference group: Non-shift workers. BMI, body mass index.

**Figure 6:**
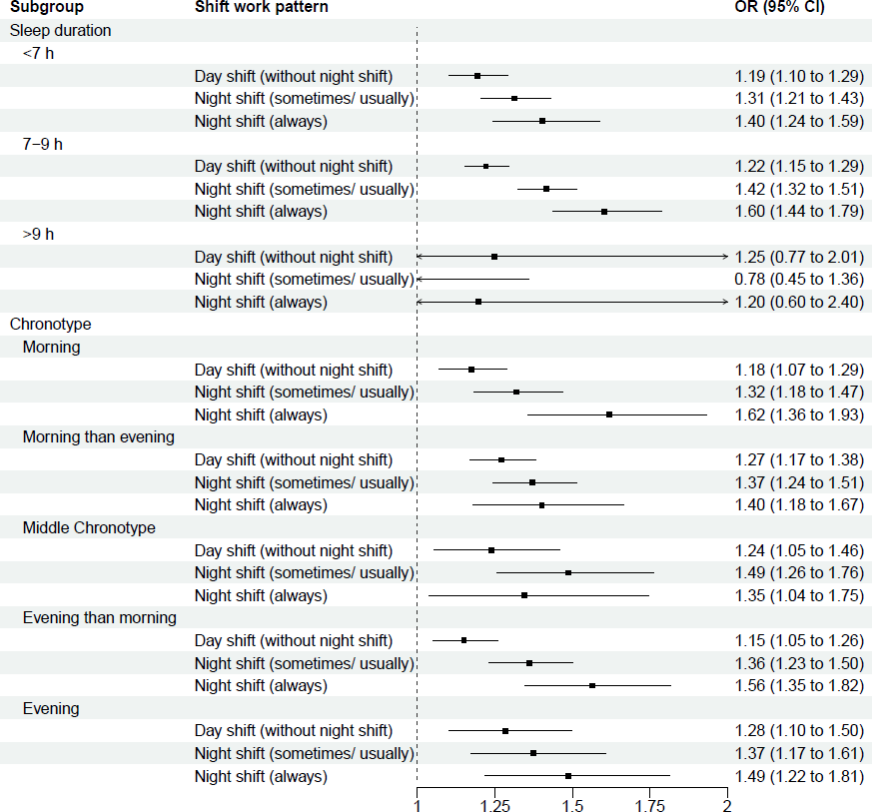
Multivariable-adjusted* associations between shift work types** and sleep disturbance, stratified by sleep traits subgroups. *Logistic regression. Adjusted for age, sex, ethnicity, education, Townsend deprivation index, household income, marital status, BMI, smoking, alcohol drinking, physical activity levels, working hours, overall health, hypertension, diabetes, sleep duration and chronotype, except for the subgroup variable itself. **Reference group: Non-shift workers. BMI, body mass index.

In the sensitivity analysis, we noted that when analyses were restricted to the same complete data across the models, findings remained unchanged. In the multivariable-adjusted regression analysis, without adjusting for sleep duration, the ORs for each shift work pattern were larger and remained significant (online supplemental table S23).

## Discussion

In this large cross-sectional study of over 280 000 employed participants, 17% of participants were shift workers and 14.8% of individuals had sleep disturbance. We found that shift work was strongly associated with an increased odds of sleep disturbance compared with non-shift (day) workers. This association was strongest for night shift workers, particularly those who reported always working nights. Notably, the relationship remained after controlling for potential confounders, including age, sex, ethnicity, education and other established risk factors. The association of shift work pattern with sleep disturbance was modified by age, ethnicity and smoking status; night shift workers were more likely to have sleep disturbance if they were younger than 55 years, from an ethnic minority background or were never smokers.

Our findings contribute to a growing body of evidence linking shift work with increased prevalence of sleep disturbance, which has been previously demonstrated in smaller-scale studies. A cross-sectional study in Belgium involving 37 662 participants, for example, found an association between shift work and both reduced sleep duration and a range of sleep disorders.^25^ A similar study conducted during the COVID-19 pandemic among 4275 Chinese nurses suggested that the risk of SWSD could be influenced by individual behaviours and the specific nature of shift work scheduling.^16^ Additionally, a longitudinal study of 1076 Norwegian nurses with a 2-year follow-up revealed that reducing night work exposure was significantly associated with lower risk of SWSD.^15^ Our study adds to these findings by providing a more comprehensive analysis of the relationship between shift work and sleep disturbance in a large population. By performing subgroup analyses, our study provides a clearer picture of the magnitude of the association across different baseline characteristics.

Increased sleep disturbance among shift workers is thought to be largely attributed to the disruption of circadian rhythms, which are critical for maintaining healthy sleep patterns.^26^ Circadian rhythms synchronise internal biological processes with external environmental cues, known as Zeitgebers (eg, light-dark cycles, meal timing and physical activity).^27^ However, shift workers are often exposed to artificial light and work during night hours, which disrupts the natural alignment between the internal clock and external cues, a phenomenon known as circadian misalignment.^27^ This misalignment, particularly among night shift workers, leads to a mismatch between their sleep-wake behaviours and biological rhythms, which has been widely linked to a variety of sleep disorders.^27 28^

Our study found that the odds of sleep disturbance were higher in individuals who always worked night shifts compared with those who worked night shifts sometimes or usually. While it might seem intuitive that irregular night shifts would cause greater circadian misalignment, evidence suggests that permanent night shift workers are often less able to adapt their circadian rhythms to the night shift schedule. In fact, only a tiny minority of permanent night shift workers (<3%) exhibit adequate adjustment to night work, as measured by circadian markers such as melatonin rhythmicity.^29^ This suggests that chronic exposure to night shifts may be particularly detrimental to sleep health, potentially contributing to long-term sleep disturbances. Circadian misalignment has been implicated not only in SWSD but also in other sleep disorders such as delayed sleep phase syndrome, non-24-hour sleep/wake disorder and recurrent hypersomnia.^30^ These disorders reflect the broader impact of disrupted biological rhythms on sleep patterns and overall health.

A key factor in circadian misalignment is chronotype, or an individual’s natural preference for morning or evening activity.^31^ We hypothesised that individuals (not just shift workers) with extreme chronotype (morning or evening types) may be more vulnerable to sleep disorders, likely due to inherent misalignment between their internal rhythms and external demands (eg, shift work or social obligations). Indeed, we demonstrate in model 3 that individuals with morning or evening chronotype were more likely to have sleep disturbance compared with those with an intermediate chronotype. However, the association between shift work and sleep disturbance was not significantly modified by chronotype, which challenges the commonly held assumption that those with an evening chronotype might be better suited to night shifts.^19 20^ Our results support those from another recent study conducted in UK Biobank which found that chronotype was not strongly associated with disordered sleep among night shift workers in the UK Biobank.^32^ These findings suggest that the effects of shift work on sleep may be more complex than simply a mismatch between shift timing and chronotype, and further investigation is needed to explore the interaction between chronotype and shift work in more detail. Longitudinal studies that assess chronotype, shift work history and tolerance, and the impact on sleep health could provide deeper insights into this relationship.

We found that night shift work was more strongly associated with sleep disturbance among individuals aged younger than 55 years, those from an ethnic minority background and never smokers. The association in older night shift workers, who may have adapted or accumulated coping strategies for long-term shift work, was weaker. On the other hand, insomnia in older adults typically occurs in the context of other medical and psychiatric disorders that are common in older age,^33^ which may result in a higher baseline risk compared with younger adults. From a sociooccupational perspective, racial and ethnic differences in shift work are evident. Black workers are more likely to have shift work with less flexibility, unpredictable schedules, higher job stress and urban living conditions, all of which may make them more vulnerable to having a sleep disorder.^34^,^36^ For smoking status, longitudinal observational evidence has identified smoking as a significant risk factor for insomnia.^37^ Additionally, a cross-sectional UK Biobank study involving 498 208 participants found that long sleep duration (>9 hours) was more common among current smokers,^38^ although the outcome definition in that study differed from ours. This evidence could indicate that current smokers have a higher baseline risk of sleep disturbance, which could attenuate the observed effect of shift work.

The strengths of our study include its large sample size, which allows for a comprehensive examination of the relationship between shift work patterns and sleep disorders across various sociodemographic groups. The UK Biobank provides high-quality, standardised data on a wide range of factors that allowed us to adjust for a broad set of confounders. This makes our study one of the largest and most diverse studies to date on this topic. Furthermore, by including participants from a more general population, our study addresses the limitations of previous research, which often focused on specific populations, such as studies on nurses, and with small sample sizes.

However, several limitations should be acknowledged. In constructing our outcome measures, we used a proxy definition of sleep disturbance based on self-reported insomnia and daytime sleepiness (or dozing) from single questionnaire items. Unlike the more comprehensive diagnostic criteria for SWSD as outlined in the ICSD-3, our study did not have data on the duration of sleep symptoms, their attribution to shift, or the ability to rule out other sleep disorders driving the complaints (eg, obstructive sleep apnoea, restless leg syndrome).

The cross-sectional nature of our study precludes any conclusions about causality. The data we analysed were collected at a single time point, which prevents us from assessing the temporal relationship between shift work exposure and the development of sleep disorders. Additionally, individuals with a tendency towards poor sleep may opt out of shift work, potentially leading to selection bias in our analysis. Longitudinal studies are needed to establish causality and examine the trajectory of sleep disturbance in relation to shift work over time. Although we controlled for a variety of potential confounders, the possibility of residual confounding—such as genetic predispositions or unmeasured health conditions—remains.

The study’s reliance on self-reported measures of sleep and the limited resolution of the shift work patterns may introduce bias^39^ or inaccuracies, particularly given that sleep patterns can be subjective and influenced by social and environmental factors. Additionally, the low participation rate (5.5%) in the UK Biobank, which is primarily composed of middle-aged, predominantly White individuals, raises concerns about selection bias and limits the generalisability of our findings to broader populations, including younger, more diverse groups. Furthermore, since our analysis used data collected between 2006 and 2010, an assessment of the associations between shift work and sleep disruption in more contemporaneous cohorts is warranted.

In conclusion, our study provides evidence that shift work is associated with a higher risk of sleep disturbance compared with non-shift work, with the greatest risk observed among those consistently working night shifts. Age, ethnicity and smoking status were found to modify the association between current shift work patterns and sleep disturbance. These findings reinforce the classification of shift work as an occupational health hazard and suggest that interventions aimed at minimising the impact of shift work on sleep health should be a priority for public health initiatives.

## Supplementary material

10.1136/bmjopen-2025-102976 online supplemental file 1

10.1136/bmjopen-2025-102976 online supplemental file 2

10.1136/bmjopen-2025-102976 online supplemental file 3

## Data Availability

Data may be obtained from a third party and are not publicly available.
