# Supplementary material for: Associations between shift work patterns and sleep disturbance: an analysis of cross-sectional data from UK Biobank
Source: BMJ Open. 2026 Jan 21;16(1):e102976. doi: 10.1136/bmjopen-2025-102976 (PMC12829381; doi:10.1136/bmjopen-2025-102976)
Supplement: online supplemental file 1 [file bmjopen-16-1-s001.docx]

**Study protocol**

**Shift work and sleep disorder: a UK Biobank cross-sectional study**

Xianqi Li, David W. Ray, Karl Smith-Byrne, Ruth Clare Travis and Rebecca C. Richmond

**1. Introduction**

To investigate associations between shift work patterns and sleep disorder and to identify the moderators.

**2. Study design and data set**

UK Biobank, cross sectional design

**3. Confirmation of main exposure and main outcome**

3.1 Main exposure: shift work

In UK Biobank, participants were asked about their employment status (paid employment, self-employed, or other). Those who were employed were further asked if their work involved shift work. Participants who indicated "sometimes," "usually," or "always" were then asked whether this included night shifts. . For both questions, response options were "never/rarely," "sometimes," "usually," or "always," with additional options for "prefer not to answer" and "do not know." The participants who selected “prefer not to answer” or “do not know” were excluded from further analysis. Based on those two questions, we categorized participants’ current shift work status as “Non-shift workers (never or rarely shifts)”, “Day shift workers (shift but never or rarely night shifts)” “Night shifts (sometimes/usually)” and “Night shifts (always)”.

3.2 Main outcome: sleep disorder

According to the (ICSD)-3^1^, SWSD is clinically assessed using three key questions: (a) Do you have a work schedule that sometimes overlaps with your usual sleep time? (b) If yes, does this cause insomnia and/or excessive sleepiness due to a reduced amount of sleep? (c) If yes, has this persisted for at least three months? Participants are classified as having SWSD if they respond “yes” to all three questions.

To approximate this, in the UK Biobank we obtained information on insomnia symptoms and excessive daytime sleepiness. In the UK Biobank, insomnia symptoms were assessed by asking participants, “Do you have trouble falling asleep at night or do you wake up in the middle of the night?” with responses options of “never/rarely”, “sometimes”, “usually” and “prefer not to answer”. Excessive sleepiness was accessed by asking, “How likely are you to doze off or fall asleep during the daytime when you don’t mean to (e.g. when working, reading or driving)” with responses of “never/rarely”, “sometimes”, “often”, “do not know” and “prefer not to answer”. Participants were categorised as having insomnia or sleepiness symptoms if they answered “sometimes” or “usually”/ “often” to this question, and participants were excluded if they selected “do not know” or “prefer not to answer”. In this study, we define sleep disorder as the presence of both insomnia and excessive sleepiness symptoms to increase the specificity of outcome assessment.

**4. Covariates**

We considered possible confounding factors, as suggested by literature review, which were collected via questionnaires or verbal interviews at baseline, including:

1. Sociodemographic factors: Age, sex, ethnic origin (White and non-White, including Mixed race, East Asian, South Asian, Black and Others), education (above A levels, A levels, below A levels), neighbourhood-level socioeconomic status as measured by the Townsend index of deprivation, household income group (less than 18000 GBP, 18000 to 30999, 31000 to 51999, 52000 to 100000, and greater than 100000) and marital status (living with partner or not).
2. Anthropometric and lifestyle factors: Body mass index (BMI; calculated as weight in kilograms divided by height in meters squared), smoking (never, former and current), alcohol intake (daily, 1-4 times a week, sometimes, never/special occasion), physical activity (low, moderate and high), working hours (more than 40 hours or not).
3. Medical conditions: self-reported overall health (poor, fair and excellent/good), self-reported hypertension and diabetes mellitus. The latter two were defined by asking if they had been told by a doctor that they had certain medical conditions.
4. Other sleep-related characteristics: Participants self-reported sleep traits on a touch-screen questionnaire at baseline, which include sleep duration (short <7h, normal 7-9h and long>9h)^2^, getting up in the morning (levels of difficulties), morning or evening person (definite morning, intermediate morning, do not know, intermediate evening, definite evening), nap during the day (never, sometimes, usually) and snoring (yes or no). For chronotype, responses of “Do not know” were included, while “Prefer not to answer” were set as missing. For all other variables, responses of “Do not know” and “Prefer not to answer” were set as missing. We include each of the sleep traits in the baseline characteristic description but only include sleep duration and chronotype as confounding factors in the regression analysis. The reasons are: 1) night shift workers may not wake up in the morning, 2) they are more likely to sleep during the day, and 3) snoring could be a symptom or a consequence of a sleep disorder rather than a contributing factor.

**5. Statistical analysis**

5.1 Baseline description

Prior to performing analyses to investigate the associations between shift work pattern and sleep disorder, we propose that descriptive and exploratory analyses will be performed to identify and display differences in baseline characteristics between: (1) different shift work patterns (2) sleep disorder or not.

5.2 Multiple logistic regression

Model 1 adjusted for age and sex; Model 2 adjusted for age, sex, ethnicity, and education; and Model 3 further adjusted for additional sociodemographic factors (Townsend deprivation index, and marital status), anthropometric and lifestyle factors (BMI, smoker, alcohol, physical activity and working hours), medical conditions (overall health, hypertension and diabetes), and sleep traits (sleep duration and chronotype). All the models were conducted using complete data.

5.3 Interaction analysis

To assess whether each covariate in Model 3 could modified the adjusted association between shift work pattern and sleep disorder, we used a log likelihood ratio test to compare models with and without cross-product interaction terms; corresponding p values were based on Chi-square statistics. Stratified analyses were conducted accordingly, stratifying by each covariate in Model 3. BMI (kg/m²) was categorized into three groups: <25, 25–30, and >30.

Analyses were conducted with Stata version 18.

**Reference**

1. American Academy of Sleep Medicine. International classification of sleep disorders. 3rd ed. Darien, IL: American Academy of Sleep Medicine 2014.

2. Hirshkowitz M, Whiton K, Albert SM, et al. National Sleep Foundation's updated sleep duration recommendations: final report. *Sleep Health* 2015;1(4):233-43. doi: 10.1016/j.sleh.2015.10.004 [published Online First: 20151031]
