# Supplementary material for: Associations between shift work patterns and sleep disturbance: an analysis of cross-sectional data from UK Biobank
Source: BMJ Open. 2026 Jan 21;16(1):e102976. doi: 10.1136/bmjopen-2025-102976 (PMC12829381; doi:10.1136/bmjopen-2025-102976)
Supplement: online supplemental file 3 [file bmjopen-16-1-s003.docx]

**Supplementary tables**

### **Table S1** UK Biobank participants’ characteristics by current shift work exposure (N=285175)

| **N (%)** | | **Non-shift workers (n=236200)** | **Shift workers (n=48975)** | | |
| --- | --- | --- | --- | --- | --- |
|  |  |  | **Day shift workers (n=24062)** | **Night shift (sometimes/ usually) (n=17940)** | **Night shift (always) (n=6973)** |
| **Sociodemographic factors** | |  |  |  |  |
| Age (mean/SD) | | 52.94(7.10) | 52.52(7.04) | 51.17(6.829) | 51.48(6.86) |
| Age group | |  |  |  |  |
|  | <45 | 35605 (15.07) | 3850 (16) | 3631 (20.24) | 1323 (18.97) |
|  | 45-49 | 45831 (19.4) | 5006 (20.8) | 4389 (24.46) | 1651 (23.68) |
|  | 50-54 | 51986 (22.01) | 5484 (22.79) | 4151 (23.14) | 1581 (22.67) |
|  | 55-60 | 53709 (22.74) | 5109 (21.23) | 3269 (18.22) | 1314 (18.84) |
|  | 61-64 | 41904 (17.74) | 3970 (16.5) | 2218 (12.36) | 1003 (14.38) |
|  | >65 | 7165 (3.03) | 643 (2.67) | 282 (1.57) | 101 (1.45) |
| Sex |  |  |  |  |  |
|  | Female | 126210 (53.43) | 12621 (52.45) | 6756 (37.66) | 2709 (38.85) |
|  | Male | 109990 (46.57) | 11441 (47.55) | 11184 (62.34) | 4264 (61.15) |
| Ethnicity |  |  |  |  |  |
|  | White | 224349 (95.24) | 21839 (91.07) | 15683 (87.72) | 6106 (87.87) |
|  | Mixed race | 1533 (0.65) | 218 (0.91) | 172 (0.96) | 59 (0.85) |
|  | East Asian | 790 (0.34) | 109 (0.45) | 81 (0.45) | 44 (0.63) |
|  | South Asian | 3993 (1.70) | 811 (3.38) | 655 (3.66) | 219 (3.15) |
|  | Black | 3276 (1.39) | 636 (2.65) | 872 (4.88) | 379 (5.45) |
|  | Other | 1611 (0.68) | 368 (1.53) | 416 (2.33) | 142 (2.04) |
|  | Missing | 648 (0.27) | 81 (0.34) | 61 (0.34) | 24 (0.34) |
| Education | |  |  |  |  |
|  | Prof Q/NVQ/HND/HNC/Degree or other professional qualification | 159542 (75.28) | 14724 (72.38) | 11638 (75.33) | 4026 (70.71) |
|  | A levels | 14150 (6.68) | 1233 (6.06) | 798 (5.17) | 278 (4.88) |
|  | O level/GSE/CSE | 38238 (18.04) | 4387 (21.56) | 3013 (19.5) | 1390 (24.41) |
|  | Missing | 24270 (10.28) | 3718 (15.45) | 2491 (13.89) | 1279 (18.34) |
| Townsend deprivation index | |  |  |  |  |
|  | Lower tertile | 82397 (34.88) | 62246 (25.96) | 4660 (25.98) | 1645 (23.59) |
|  | Middle tertile | 79808 (33.79) | 7508 (31.20) | 5439 (30.32) | 2150 (30.83) |
|  | Upper tertile | 73682 (31.19) | 10262 (42.65) | 7813 (43.55) | 3161 (45.33) |
|  | Missing | 312 (0.13) | 46 (0.19) | 28 (0.16) | 17 (0.24) |
| Household income | |  |  |  |  |
|  | Less than 18,000 GBP | 19990 (9.39) | 3775 (17.82) | 2061 (12.81) | 971 (15.57) |
|  | 18,000 to 30,999 | 43902 (20.63) | 6378 (30.10) | 4403 (27.37) | 1932 (30.98) |
|  | 31000 to 51999 | 66525 (31.26) | 6494 (30.65) | 5520 (34.32) | 2254 (36.14) |
|  | 52000 to 100000 | 64696 (30.40) | 3881 (18.32) | 3447 (21.43) | 997 (15.99) |
|  | Greater than 100000 | 17698 (8.32) | 661 (3.12) | 654 (4.07) | 83 (1.33) |
|  | Missing | 23389 (9.90) | 2873 (11.94) | 1855 (10.34) | 736 (10.55) |
| Marital status | |  |  |  |  |
|  | Living with partner | 176669 (74.98) | 16321 (68.06) | 12237 (68.5) | 4706 (67.85) |
|  | Not living with partner | 58943 (25.02) | 7658 (31.94) | 5628 (31.5) | 2230 (32.15) |
|  | Missing | 588 (0.25) | 83 (0.34) | 75 (0.42) | 37 (0.53) |
| **Anthropometric and lifestyle factors** | |  |  |  |  |
| BMI groups | |  |  |  |  |
|  | <18.5 (underweight) | 1139 (0.48) | 105 (0.44) | 61 (0.34) | 24 (0.35) |
|  | 18.5-24.9 (healthy) | 83283 (35.38) | 7230 (30.19) | 4601 (25.8) | 1615 (23.25) |
|  | 25-29.9 (overweight) | 98937 (42.03) | 10042 (41.93) | 7823 (43.87) | 3102 (44.67) |
|  | 30-39.9 (obese) | 48296 (20.52) | 5978 (24.96) | 4891 (27.43) | 2010 (28.94) |
|  | 40+ (severely obese) | 3726 (1.58) | 596 (2.49) | 455 (2.55) | 194 (2.79) |
|  | Missing | 819 (0.35) | 111 (0.46) | 109 (0.61) | 28 (0.40) |
| BMI (mean/SD) | | 27.09 (4.64) | 27.79 (4.99) | 28.21 (4.91) | 28.51 (4.88) |
| Smoker |  |  |  |  |  |
|  | Never | 137225 (58.23) | 12868 (53.65) | 9469 (53) | 3625 (52.1) |
|  | Former | 75412 (32) | 7768 (32.39) | 5486 (30.71) | 2103 (30.22) |
|  | Current | 23004 (9.76) | 3350 (13.97) | 2910 (16.29) | 1230 (17.68) |
|  | Missing | 559 (0.24) | 76 (0.32) | 75 (0.42) | 15 (0.22) |
| Alcohol |  |  |  |  |  |
|  | Daily | 48426 (20.51) | 4072 (16.93) | 2882 (16.08) | 720 (10.34) |
|  | 1-4 times/week | 124974 (52.94) | 11631 (48.37) | 8960 (49.98) | 3454 (49.59) |
|  | Sometimes | 27227 (11.53) | 3159 (13.14) | 2354 (13.13) | 1050 (15.08) |
|  | Never/special occasion | 35460 (15.02) | 5185 (21.56) | 3730 (20.81) | 1741 (25) |
|  | Missing | 113 (0.05) | 15 (0.06) | 14 (0.08) | 8 (0.11) |
| Physical activity | |  |  |  |  |
|  | Low | 41218 (20.94) | 2948 (15.6) | 2017 (14.35) | 655 (12.54) |
|  | Moderate | 81763 (41.54) | 6583 (34.84) | 4496 (32) | 1537 (29.43) |
|  | High | 73853 (37.52) | 9362 (49.55) | 7539 (53.65) | 3030 (58.02) |
|  | Missing | 39366 (16.67) | 5169 (21.48) | 3888 (21.67) | 1751 (25.11) |
| Working hours | |  |  |  |  |
|  | <=40 | 175912 (74.48) | 17856 (74.21) | 10950 (61.04) | 4242 (60.83) |
|  | >40 | 60288 (25.52) | 6206 (25.79) | 6990 (38.96) | 2731 (39.17) |
| **Medical conditions** | |  |  |  |  |
| Overall health | |  |  |  |  |
|  | Poor | 4873 (2.07) | 695 (2.9) | 582 (3.26) | 247 (3.56) |
|  | Fair | 42181 (17.9) | 5563 (23.22) | 4419 (24.77) | 1776 (25.61) |
|  | Excellent/good | 188555 (80.03) | 17699 (73.88) | 12838 (71.97) | 4912 (70.83) |
|  | Missing | 591 (0.25) | 105 (0.44) | 101 (0.56) | 38 (0.54) |
| Hypertension | |  |  |  |  |
|  | Yes | 49090 (20.78) | 5450 (22.65) | 4081 (22.75) | 1646 (23.61) |
|  | No | 187104 (79.22) | 18611 (77.35) | 13859 (77.25) | 5327 (76.39) |
|  | Missing | 6 (0) | 1 (0) | 0 (0) | 0 (0) |
| Diabetes |  |  |  |  |  |
|  | Yes | 7941 (3.37) | 1059 (4.42) | 856 (4.79) | 333 (4.8) |
|  | No | 227858 (96.63) | 22924 (95.58) | 17010 (95.21) | 6599 (95.20) |
|  | Missing | 401 (0.17) | 79 (0.33) | 74 (0.41) | 41 (0.59) |
| **Sleep-related characteristics** | |  |  |  |  |
| Sleep duration | |  |  |  |  |
|  | <7 | 57222 (24.28) | 7130 (29.78) | 6070 (34.04) | 2843 (41.24) |
|  | 7-9 | 176835 (75.04) | 16552 (69.14) | 11553 (64.79) | 3933 (57.06) |
|  | >9 | 1599 (0.68) | 258 (1.08) | 209 (1.17) | 117 (1.7) |
|  | Missing | 544 (0.23) | 122 (0.51) | 108 (0.60) | 80 (1.15) |
| Difficulty in getting up in the morning | |  |  |  |  |
|  | Not at all easy | 8325 (3.56) | 980 (4.11) | 779 (4.38) | 346 (5.06) |
|  | Not very easy | 36904 (15.76) | 3615 (15.16) | 2696 (15.17) | 1022 (14.95) |
|  | Fairly easy | 124835 (53.31) | 12342 (51.77) | 8983 (50.56) | 3346 (48.95) |
|  | Very easy | 64095 (27.37) | 6901 (28.95) | 5309 (29.88) | 2121 (31.03) |
|  | Missing | 2041 (0.86) | 224 (0.93) | 173 (0.96) | 138 (1.98) |
| Chronotype | |  |  |  |  |
|  | Morning | 54638 (23.35) | 6078 (25.54) | 4059 (22.84) | 1343 (19.51) |
|  | Morning than evening | 76397 (32.65) | 7226 (30.37) | 4897 (27.56) | 1462 (21.24) |
|  | Middle | 23114 (9.88) | 2330 (9.79) | 2092 (11.77) | 927 (13.47) |
|  | Evening than morning | 61021 (26.08) | 6281 (26.39) | 4966 (27.95) | 1995 (28.98) |
|  | Evening | 18786 (8.03) | 1882 (7.91) | 1756 (9.88) | 1156 (16.8) |
|  | Missing | 2244 (0.95) | 265 (1.10) | 170 (0.95) | 90 (1.29) |
| Nap during day | |  |  |  |  |
|  | Never/rarely | 151829 (64.31) | 12908 (53.79) | 8802 (49.16) | 2823 (40.7) |
|  | Sometimes | 76735 (32.5) | 9918 (41.33) | 8219 (45.91) | 3445 (49.67) |
|  | Usually | 7537 (3.19) | 1172 (4.88) | 883 (4.93) | 668 (9.63) |
|  | Missing | 99 (0.04) | 64 (0.27) | 36 (0.20) | 37 (0.53) |
| Sleeplessness / insomnia | |  |  |  |  |
|  | Never/rarely | 65679 (27.81) | 6166 (25.63) | 5030 (28.04) | 1938 (27.79) |
|  | Sometimes | 112718 (47.72) | 11598 (48.2) | 8709 (48.55) | 3261 (46.77) |
|  | Usually | 57803 (24.47) | 6298 (26.17) | 4201 (23.42) | 1774 (25.44) |
| Snoring |  |  |  |  |  |
|  | Yes | 83933 (37.83) | 8926 (40.31) | 7221 (43.49) | 2757 (42.92) |
|  | No | 137936 (62.17) | 13218 (59.69) | 9384 (56.51) | 3667 (57.08) |
|  | Missing | 14331 (6.07) | 1918 (7.97) | 1335 (7.44) | 549 (7.87) |
| Daytime dozing | |  |  |  |  |
|  | Never/rarely | 192713 (81.59) | 18472 (76.77) | 13339 (74.35) | 4891 (70.14) |
|  | Sometimes | 39029 (16.52) | 4952 (20.58) | 4084 (22.76) | 1802 (25.84) |
|  | Often | 4447 (1.88) | 636 (2.64) | 516 (2.88) | 278 (3.99) |
|  | All of the time | 11 (0) | 2 (0.01) | 1 (0.01) | 2 (0.03) |
| Sleep disruption^*^ | |  |  |  |  |
|  | Yes | 33012 (13.98) | 4366 (18.14) | 3530 (19.68) | 1573 (22.56) |
|  | No | 203188 (86.02) | 19696 (81.86) | 14410 (80.32) | 5400 (77.44) |

* Sleep disruption was defined as both insomnia and excessive sleepiness.

### **Table S2 Baseline characteristics by sleep disturbance**

| **Baseline characteristics (n=285175)** | | | **No sleep disturbance (n=242694)** | | **Sleep disturbance**  **(n= 42481)** | |
| --- | --- | --- | --- | --- | --- | --- |
|  |  |  |  |  |  |  |
| **Sociodemographic factors** | | | N | % | N | % |
| Age (mean/SD) | | | 52.51 | 7.08 | 54.21 | 6.98 |
| Age group | | |  |  |  |  |
|  | | <45 | 39839 | 16.42 | 4570 | 10.76 |
|  | | 45-49 | 49821 | 20.53 | 7056 | 16.61 |
|  | | 50-54 | 53919 | 22.22 | 9283 | 21.85 |
|  | | 55-60 | 52741 | 21.73 | 10660 | 25.09 |
|  | | 61-64 | 39898 | 16.44 | 9197 | 21.65 |
|  | | >65 | 6476 | 2.67 | 1715 | 4.04 |
| Sex | |  |  |  |  |  |
|  | | Female | 126624 | 52.17 | 21672 | 51.02 |
|  | | Male | 116070 | 47.83 | 20809 | 48.98 |
| Ethnicity | |  |  |  |  |  |
|  | | White | 229662 | 94.89 | 38315 | 90.52 |
|  | | Non-white | 12373 | 5.11 | 4011 | 9.48 |
| Ethnicity |  | |  |  |  |  |
|  | White | | 229662 | 94.89 | 38315 | 90.52 |
|  | Mixed race | | 1646 | 0.68 | 336 | 0.79 |
|  | East Asian | | 770 | 0.32 | 254 | 0.60 |
|  | South Asian | | 4266 | 1.76 | 1412 | 3.34 |
|  | Black | | 3782 | 1.56 | 1381 | 3.26 |
|  | Other | | 1909 | 0.79 | 628 | 1.48 |
| Education | | |  |  |  |  |
|  | | Prof Q/NVQ/HND/HNC/Degree or other professional qualification | 162957 | 75.01 | 26973 | 74.6 |
|  | | A levels | 14255 | 6.56 | 2204 | 6.1 |
|  | | O level/GSE/CSE | 40048 | 18.43 | 6980 | 19.3 |
| Townsend deprivation index (mean/SD) | | | -1.41 | 2.97 | -0.98 | 3.18 |
| Household income | | |  |  |  |  |
|  | | Less than 18,000 | 21187 | 9.69 | 5610 | 14.87 |
|  | | 18,000 to 30,999 | 46939 | 21.47 | 9676 | 25.64 |
|  | | 31,000 to 51,999 | 69490 | 31.79 | 11303 | 29.95 |
|  | | 52,000 to 100,000 | 63973 | 29.27 | 9048 | 23.98 |
|  | | Greater than 100,000 | 16996 | 7.78 | 2100 | 5.56 |
| Marital status | | |  |  |  |  |
|  | | Living with partner | 179452 | 74.14 | 30481 | 71.97 |
|  | | Not living with partner | 62589 | 25.86 | 11870 | 28.03 |
| **Anthropometric and lifestyle factors** | | |  |  |  |  |
| BMI groups | | |  |  |  |  |
|  | | <18.5 (underweight) | 1145 | 0.47 | 184 | 0.44 |
|  | | 18.5-24.9 (healthy) | 84344 | 34.88 | 12385 | 29.3 |
|  | | 25-29.9 (overweight) | 102240 | 42.28 | 17664 | 41.8 |
|  | | 30-39.9 (obese) | 50353 | 20.82 | 10822 | 25.61 |
|  | | 40+ (severely obese) | 3763 | 1.56 | 1208 | 2.86 |
| BMI (mean/SD) | | | 27.13 | 4.63 | 27.98 | 5.12 |
| Smoker | |  |  |  |  |  |
|  | | Never | 139341 | 57.56 | 23846 | 56.3 |
|  | | Former | 76749 | 31.7 | 14020 | 33.1 |
|  | | Current | 26008 | 10.74 | 4486 | 10.59 |
| Alcohol | |  |  |  |  |  |
|  | | Daily | 48559 | 20.02 | 7541 | 17.76 |
|  | | 1-4 times/week | 127988 | 52.76 | 21031 | 49.54 |
|  | | Sometimes | 28562 | 11.77 | 5228 | 12.31 |
|  | | Never/special occasion | 37460 | 15.44 | 8656 | 20.39 |
| Physical activity | | |  |  |  |  |
|  | | Low | 39610 | 19.7 | 7228 | 21.3 |
|  | | Moderate | 80823 | 40.2 | 13556 | 39.96 |
|  | | High | 80640 | 40.1 | 13144 | 38.74 |
| Working hours | | |  |  |  |  |
|  | | <=40 | 176851 | 72.87 | 32109 | 75.58 |
|  | | >40 | 65843 | 27.13 | 10372 | 24.42 |
| **Medical conditions** | | |  |  |  |  |
| Overall health | | |  |  |  |  |
|  | | Poor | 4461 | 1.84 | 1936 | 4.58 |
|  | | Fair | 42806 | 17.68 | 11133 | 26.34 |
|  | | Excellent/good | 194812 | 80.47 | 29192 | 69.08 |
| Hypertension | | Yes | 49096 | 20.23 | 11171 | 26.3 |
| Diabetes | | Yes | 7849 | 3.24 | 2340 | 5.53 |
| **Sleep-related characteristics** | | |  |  |  |  |
| Sleep duration | | |  |  |  |  |
|  | | <7 | 58632 | 24.22 | 14633 | 34.66 |
|  | | 7-9 | 181782 | 75.08 | 27091 | 64.17 |
|  | | >9 | 1688 | 0.7 | 495 | 1.17 |
| Difficulty in getting up in the morning | | |  |  |  |  |
|  | | Not at all easy | 8005 | 3.33 | 2425 | 5.76 |
|  | | Not very easy | 36125 | 15.02 | 8112 | 19.27 |
|  | | Fairly easy | 128194 | 53.3 | 21312 | 50.63 |
|  | | Very easy | 68180 | 28.35 | 10246 | 24.34 |
| Chronotype | | |  |  |  |  |
|  | | morning | 55673 | 23.17 | 10445 | 24.81 |
|  | | morning than evening | 76725 | 31.93 | 13257 | 31.49 |
|  | | Middle | 24441 | 10.17 | 4022 | 9.55 |
|  | | evening than morning | 63543 | 26.44 | 10720 | 25.47 |
|  | | evening | 19928 | 8.29 | 3652 | 8.68 |
| Nap during day | | |  |  |  |  |
|  | | Never/rarely | 162269 | 66.9 | 14093 | 33.25 |
|  | | Sometimes | 73115 | 30.14 | 25202 | 59.45 |
|  | | Usually | 7166 | 2.95 | 3094 | 7.3 |
| Sleeplessness / insomnia | | |  |  |  |  |
|  | | Never/rarely | 78813 | 32.47 | 0 | 0 |
|  | | Sometimes | 109929 | 45.3 | 26357 | 62.04 |
|  | | Usually | 53952 | 22.23 | 16124 | 37.96 |
| Snoring | | Yes | 84434 | 37.09 | 18403 | 46.69 |
| Daytime dozing | | |  |  |  |  |
|  | | Never/rarely | 229415 | 94.53 | 0 | 0 |
|  | | Sometimes | 11918 | 4.91 | 37949 | 89.33 |
|  | | Often | 1357 | 0.56 | 4520 | 10.64 |
|  | | All of the time | 4 | 0 | 12 | 0.03 |
| **Shift work** | | |  |  |  |  |
|  | | dayworkers (non-shift) | 203188 | 83.72 | 33012 | 77.71 |
|  | | day shift | 19696 | 8.12 | 4366 | 10.28 |
|  | | night shift (sometimes/usually) | 14410 | 5.94 | 3530 | 8.31 |
|  | | night shift (always) | 5400 | 2.23 | 1573 | 3.7 |

# **Table S3 Assessment of covariates for multicollinearity through Variance Inflation Factors**

| **Variable** | | **Variance inflation factor (VIF)** |
| --- | --- | --- |
| **Sociodemographic factors** | |  |
| Age group (versus <45) | |  |
|  | 45-49 | 1.77 |
|  | 50-54 | 1.82 |
|  | 55-60 | 1.83 |
|  | 61-64 | 1.71 |
|  | >65 | 1.15 |
| Sex (versus female) | |  |
|  | Male | 1.18 |
| Ethnicity (versus white) | |  |
|  | Mixed race | 1.01 |
|  | East Asian | 1.01 |
|  | South Asian | 1.04 |
|  | Black | 1.05 |
|  | Other | 1.01 |
| Education (versus Prof Q/NVQ/HND/HNC/Degree or other professional qualification ) | |  |
|  | A levels | 1.02 |
|  | O level/GSE/CSE | 1.07 |
| Townsend deprivation index (versus quantile 1) | |  |
|  | quantile 2 | 1.32 |
|  | quantile 3 | 1.44 |
| Household income (versus to less than 18,000 GBP) | |  |
|  | 18,000 to 30,999 | 2.90 |
|  | 31,000 to 51,999 | 3.90 |
|  | 52,000 to 100,000 | 4.31 |
|  | Greater than 100,000 | 2.35 |
| Marital status (versus Living with partner ) | |  |
|  | Not living with partner | 1.27 |
| **Anthropometric and lifestyle factors** | |  |
| BMI |  | 1.18 |
| Smoker (versus never) | |  |
|  | former | 1.11 |
|  | current | 1.12 |
| Alcohol (versus daily) | |  |
|  | 1-4 times/week | 1.64 |
|  | Sometimes | 2.45 |
|  | Never/special occasion | 2.22 |
| Physical activity (versus low) | |  |
|  | Moderate | 1.82 |
|  | High | 1.89 |
| Working hours (versus <=40) | |  |
|  | >40 | 1.16 |
| Overall health (versus poor) | |  |
|  | Fair | 8.01 |
|  | Excellent/good | 8.32 |
| Hypertension | |  |
|  | Yes | 1.14 |
| Diabetes |  |  |
|  | Yes | 1.07 |
| **Sleep-related characteristics** | |  |
| Sleep duration (versus <7h) | |  |
|  | 7-9 | 1.04 |
|  | >9 | 1.01 |
| Chronotype (versus middle) | |  |
|  | morning | 2.77 |
|  | morning than evening | 3.16 |
|  | evening than morning | 2.94 |
|  | evening | 1.83 |
| **Shiftwork** | |  |
| Shift workers (versus non-shift workers) | |  |
|  | day shift | 1.03 |
|  | night shift (sometimes/usually) | 1.04 |
|  | night shift (always) | 1.03 |
| **Mean VIF** | | 1.98 |

### **Table S4 Adjusted association* of shift work patterns and sleep disruption stratified by sex**

|  |  | Female (n=99629) | | Male (n=97776) | | |
| --- | --- | --- | --- | --- | --- | --- |
|  |  | OR (95% CI) | *p* value | OR (95% CI) | *p* value | |
| Shift workers | |  |  |  | |  |
|  | non-shift workers | 1.00 (Reference) |  | 1.00 (Reference) | |  |
|  | day shift | 1.15 (1.08, 1.23) | <0.0001 | 1.27 (1.19, 1.36) | | <0.0001 |
|  | night shift (sometimes/usually) | 1.30 (1.19, 1.41) | <0.0001 | 1.41 (1.32, 1.51) | | <0.0001 |
|  | night shift (always) | 1.56 (1.37, 1.77) | <0.0001 | 1.46 (1.32, 1.63) | | <0.0001 |
| *p* for interaction^**^: 0.0784 | | | | | | |

* Adjusted for all covariates: age groups, sex, education, Townsend deprivation index, household income, marital status, BMI index, smoking status, alcohol, physical activity, working hours, overall health, hypertension, diabetes, sleep durations and chronotypes.

** Interaction were tested using a log-likelihood ratio test to compare logistic regression models with and without cross-product interaction terms, adjusted all other covariates.

### **Table S5 Adjusted association of shift work patterns and sleep disruption stratified by age**

|  |  | 55+ (n=75910) | | <55 (n=121495) | |
| --- | --- | --- | --- | --- | --- |
|  |  | OR (95% CI) | *p* value | OR (95% CI) | *p* value |
| Shift workers | |  |  |  |  |
|  | non-shift workers | 1.00 (Reference) |  | 1.00 (Reference) |  |
|  | day shift | 1.16 (1.08, 1.24) | <0.0001 | 1.24 (1.17, 1.32) | <0.0001 |
|  | night shift (sometimes/usually) | 1.31 (1.20, 1.44) | <0.0001 | 1.37 (1.28, 1.46) | <0.0001 |
|  | night shift (always) | 1.20 (1.04, 1.39) | 0.0130 | 1.63 (1.48, 1.80) | <0.0001 |
| *p* for interaction: <0.0001 | | | | | |

### **Table S6 Adjusted association of shift work patterns and sleep disruption stratified by ethnicity**

|  |  | White (n=186968) | | Non-White (n=10437) | |
| --- | --- | --- | --- | --- | --- |
|  |  | OR (95% CI) | *p* value | OR (95% CI) | *p* value |
| Shift workers | |  |  |  |  |
|  | non-shift workers | 1.00 (Reference) |  | 1.00 (Reference) |  |
|  | day shift | 1.20 (1.15, 1.26) | <0.0001 | 1.29 (1.11, 1.50) | 0.001 |
|  | night shift (sometimes/usually) | 1.33 (1.26, 1.41) | <0.0001 | 1.61 (1.40, 1.85) | <0.0001 |
|  | night shift (always) | 1.44 (1.32, 1.57) | <0.0001 | 1.92 (1.55, 2.36) | <0.0001 |
| *p* for interaction: 0.0005 | | | | | |

### **Table S7 Adjusted association of shift work patterns and sleep disruption by ethnicity (in participants with different non-white ethnic background)**

|  |  | Mixed Race (n= 1393) | | East Asian (n=658) | | South Asian (n=3479) | | Black or Black British (n=3301) | | Other (n=1606) | |
| --- | --- | --- | --- | --- | --- | --- | --- | --- | --- | --- | --- |
|  |  | OR (95% CI) | *p* value | OR (95% CI) | *p* value | OR (95% CI) | *p* value | OR (95% CI) | *p* value | OR (95% CI) | *p* value |
| Shift workers | | |  |  |  |  |  |  |  |  |  |
|  | non-shift workers | 1.00 (Reference) |  | 1.00 (Reference) |  | 1.00 (Reference) |  | 1.00 (Reference) |  | 1.00 (Reference) |  |
|  | day shift | 1.39 (0.86, 2.25) | 0.1720 | 0.88 (0.46, 1.67) | 0.6880 | 1.47 (1.14, 1.88) | 0.0030 | 1.27 (0.97, 1.66) | 0.0820 | 1.26 (0.86, 1.84) | 0.2430 |
|  | night shift (sometimes/usually) | 1.30 (0.74, 2.27) | 0.3610 | 0.70 (0.32, 1.55) | 0.3820 | 1.58 (1.21, 2.05) | 0.0010 | 1.73 (1.39, 2.16) | <0.0001 | 1.80 (1.28, 2.54) | 0.0010 |
|  | night shift (always) | 1.33 (0.51, 3.46) | 0.5590 | 1.00 (0.37, 2.70) | 1.0000 | 1.94 (1.27, 2.98) | 0.0020 | 1.91 (1.40, 2.61) | <0.0001 | 2.05 (1.20, 3.52) | 0.0090 |
| *p* for interaction: 0.0200 | | | | | | | | | | | |

### **Table S8 Adjusted association of shift work patterns and sleep disruption stratified by education**

|  |  | Prof Q/NVQ/HND/HNC/Degree or other professional qualification  (n= 150532) | | A levels (n= 13121) | | O level/GSE/CSE (n= 33752) | |
| --- | --- | --- | --- | --- | --- | --- | --- |
|  |  | OR (95% CI) | p value | OR (95% CI) | p value | OR (95% CI) | p value |
| Shift workers | | |  |  |  |  |  |
|  | non-shift workers | 1.00 (Reference) |  | 1.00 (Reference) |  | 1.00 (Reference) |  |
|  | day shift | 1.17 (1.11, 1.24) | <0.0001 | 1.38 (1.14, 1.66) | 0.0010 | 1.33 (1.20, 1.47) | <0.0001 |
|  | night shift (sometimes/usually) | 1.34 (1.26, 1.42) | <0.0001 | 1.55 (1.24, 1.95) | <0.0001 | 1.47 (1.31, 1.66) | <0.0001 |
|  | night shift (always) | 1.50 (1.36, 1.65) | <0.0001 | 1.31 (0.89, 1.91) | 0.1690 | 1.56 (1.31, 1.85) | <0.0001 |
| *p* for interaction: 0.0618 | | | | | | | |

### **Table S9 Adjusted association of shift work patterns and sleep disruption stratified by Townsend deprivation index**

|  |  | Quantile 1, less deprivation  (n= 68817) | | Quantile 2 (n= 66301) | | Quantile 3 (n= 62287) | |
| --- | --- | --- | --- | --- | --- | --- | --- |
|  |  | OR (95% CI) | p value | OR (95% CI) | p value | OR (95% CI) | p value |
| Shift workers | | |  |  |  |  |  |
|  | non-shift workers | 1.00 (Reference) |  | 1.00 (Reference) |  | 1.00 (Reference) |  |
|  | day shift | 1.26 (1.16, 1.38) | <0.0001 | 1.22 (1.12, 1.32) | <0.0001 | 1.17 (1.09, 1.26) | <0.0001 |
|  | night shift (sometimes/usually) | 1.33 (1.20, 1.48) | <0.0001 | 1.33 (1.21, 1.46) | <0.0001 | 1.40 (1.30, 1.52) | <0.0001 |
|  | night shift (always) | 1.31 (1.10, 1.56) | 0.0030 | 1.46 (1.26, 1.69) | <0.0001 | 1.61 (1.43, 1.81) | <0.0001 |
| *p* for interaction: 0.1339 | | | | | | | |

### **Table S10 Adjusted association of shift work patterns and sleep disruption stratified by household income levels**

|  |  | less than 18,000 GBP (n=15512) | | 18,000 to 30,999 (n=38651) | | 31,000 to 51,999 (n=63449) | | 52,000 to 100,000 (n=62620) | | Greater than 100,000 (n=17173) | |
| --- | --- | --- | --- | --- | --- | --- | --- | --- | --- | --- | --- |
|  |  | OR (95% CI) | *p* value | OR (95% CI) | *p* value | OR (95% CI) | *p* value | OR (95% CI) | *p* value | OR (95% CI) | *p* value |
| Shift workers | | |  |  |  |  |  |  |  |  |  |
|  | non-shift workers | 1.00 (Reference) |  | 1.00 (Reference) |  | 1.00 (Reference) |  | 1.00 (Reference) |  | 1.00 (Reference) |  |
|  | day shift | 1.32 (1.18, 1.48) | <0.0001 | 1.24 (1.14, 1.36) | <0.0001 | 1.18 (1.08, 1.28) | <0.0001 | 1.15 (1.03, 1.28) | 0.0120 | 1.22 (0.95, 1.57) | 0.1140 |
|  | night shift (sometimes/usually) | 1.47 (1.26, 1.71) | <0.0001 | 1.41 (1.28, 1.56) | <0.0001 | 1.36 (1.25, 1.49) | <0.0001 | 1.31 (1.17, 1.46) | <0.0001 | 1.17 (0.90, 1.52) | 0.2320 |
|  | night shift (always) | 1.55 (1.26, 1.91) | <0.0001 | 1.64 (1.41, 1.90) | <0.0001 | 1.44 (1.26, 1.65) | <0.0001 | 1.38 (1.13, 1.69) | 0.0020 | 1.28 (0.64, 2.53) | 0.4820 |
| *p* for interaction: 0.2137 | | | | | | | | | | | |

### **Table S11 Adjusted association of shift work patterns and sleep disruption stratified by marital status**

|  |  | Living with partner (n= 146534) | | Not living with partner (n= 50871) | |
| --- | --- | --- | --- | --- | --- |
|  |  | OR (95% CI) | p value | OR (95% CI) | p value |
| Shift workers | | |  |  |  |
|  | non-shift workers | 1.00 (Reference) |  | 1.00 (Reference) |  |
|  | day shift | 1.22 (1.15, 1.29) | <0.0001 | 1.19 (1.10, 1.29) | <0.0001 |
|  | night shift (sometimes/usually) | 1.33 (1.25, 1.42) | <0.0001 | 1.44 (1.31, 1.58) | <0.0001 |
|  | night shift (always) | 1.51 (1.37, 1.67) | <0.0001 | 1.49 (1.29, 1.71) | <0.0001 |
| *p* for interaction: 0.1402 | | | | | |

### **Table S12 Adjusted association of shift work patterns and sleep disruption stratified by BMI (kg/m^2^)**

|  |  | <24.9 (n= 70568) | | 25-29.9 (n= 83434) | | >30 (n= 43403) | |
| --- | --- | --- | --- | --- | --- | --- | --- |
|  |  | OR (95% CI) | p value | OR (95% CI) | p value | OR (95% CI) | p value |
| Shift workers | | |  |  |  |  |  |
|  | non-shift workers | 1.00 (Reference) |  | 1.00 (Reference) |  | 1.00 (Reference) |  |
|  | day shift | 1.27 (1.17, 1.38) | <0.0001 | 1.18 (1.10, 1.27) | <0.0001 | 1.20 (1.10, 1.31) | <0.0001 |
|  | night shift (sometimes/usually) | 1.26 (1.13, 1.40) | <0.0001 | 1.44 (1.33, 1.55) | <0.0001 | 1.36 (1.24, 1.49) | <0.0001 |
|  | night shift (always) | 1.41 (1.18, 1.67) | <0.0001 | 1.49 (1.32, 1.69) | <0.0001 | 1.57 (1.37, 1.81) | <0.0001 |
| *p* for interaction: 1.0000 | | | | | | | |

### **Table S13 Adjusted association of shift work patterns and sleep disruption stratified by smoke status**

|  |  | Never (n= 115436) | | Former (n= 62365) | | Current (n= 19604) | |
| --- | --- | --- | --- | --- | --- | --- | --- |
|  |  | OR (95% CI) | p value | OR (95% CI) | p value | OR (95% CI) | p value |
| Shift workers | | |  |  |  |  |  |
|  | non-shift workers | 1.00 (Reference) |  | 1.00 (Reference) |  | 1.00 (Reference) |  |
|  | day shift | 1.21 (1.14, 1.29) | <0.0001 | 1.18 (1.08, 1.28) | <0.0001 | 1.32 (1.15, 1.50) | <0.0001 |
|  | night shift (sometimes/usually) | 1.36 (1.27, 1.46) | <0.0001 | 1.41 (1.28, 1.54) | <0.0001 | 1.31 (1.14, 1.51) | <0.0001 |
|  | night shift (always) | 1.66 (1.49, 1.85) | <0.0001 | 1.43 (1.23, 1.66) | <0.0001 | 1.14 (0.91, 1.41) | 0.2480 |
| *p* for interaction: 0.0370 | | | | | | | |

### **Table S14 Adjusted association of shift work patterns and sleep disruption stratified by alcohol intake frequency**

|  |  | Daily (n= 41392) | | 1-4 times/week (n=105236) | | Sometimes (n=22715) | | Never/special occasion  (n= 28062) | |
| --- | --- | --- | --- | --- | --- | --- | --- | --- | --- |
|  |  | OR (95% CI) | p value | OR (95% CI) | p value | OR (95% CI) | p value | OR (95% CI) | p value |
| Shift workers | | |  |  |  |  |  |  |  |
|  | non-shift workers | 1.00 (Reference) |  | 1.00 (Reference) |  | 1.00 (Reference) |  | 1.00 (Reference) |  |
|  | day shift | 1.17 (1.04, 1.31) | 0.0070 | 1.21 (1.13, 1.29) | <0.0001 | 1.29 (1.14, 1.47) | <0.0001 | 1.21 (1.09, 1.34) | <0.0001 |
|  | night shift (sometimes/usually) | 1.47 (1.30, 1.67) | <0.0001 | 1.39 (1.29, 1.49) | <0.0001 | 1.27 (1.10, 1.47) | 0.0020 | 1.32 (1.17, 1.48) | <0.0001 |
|  | night shift (always) | 1.37 (1.07, 1.77) | 0.0150 | 1.53 (1.36, 1.72) | <0.0001 | 1.41 (1.14, 1.73) | 0.0010 | 1.54 (1.31, 1.81) | <0.0001 |
| *p* for interaction: 0.6672 | | | | | | | | | |

### **Table S15 Adjusted association of shift work patterns and sleep disruption stratified by physical activity levels**

|  |  | Low (n= 40012) | | Moderate (n= 81228) | | High (n= 76165) | |
| --- | --- | --- | --- | --- | --- | --- | --- |
|  |  | OR (95% CI) | p value | OR (95% CI) | p value | OR (95% CI) | p value |
| Shift workers | | |  |  |  |  |  |
|  | non-shift workers | 1.00 (Reference) |  | 1.00 (Reference) |  | 1.00 (Reference) |  |
|  | day shift | 1.34 (1.20, 1.50) | <0.0001 | 1.20 (1.11, 1.29) | <0.0001 | 1.17 (1.09, 1.26) | <0.0001 |
|  | night shift (sometimes/usually) | 1.26 (1.10, 1.45) | 0.0010 | 1.40 (1.28, 1.53) | <0.0001 | 1.37 (1.27, 1.48) | <0.0001 |
|  | night shift (always) | 1.36 (1.08, 1.71) | 0.0080 | 1.50 (1.30, 1.73) | <0.0001 | 1.55 (1.39, 1.73) | <0.0001 |
| *p* for interaction: 0.3131 | | | | | | | |

### **Table S16 Adjusted association of shift work patterns and sleep disruption stratified by working hours**

|  |  | <=40 (n= 141321) | | >40 (n= 56084) | |
| --- | --- | --- | --- | --- | --- |
|  |  | OR (95% CI) | p value | OR (95% CI) | p value |
| Shift workers | | |  |  |  |
|  | non-shift workers | 1.00 (Reference) |  | 1.00 (Reference) |  |
|  | day shift | 1.22 (1.16, 1.29) | <0.0001 | 1.19 (1.08, 1.30) | <0.0001 |
|  | night shift (sometimes/usually) | 1.32 (1.24, 1.42) | <0.0001 | 1.43 (1.31, 1.56) | <0.0001 |
|  | night shift (always) | 1.49 (1.34, 1.65) | <0.0001 | 1.51 (1.32, 1.72) | <0.0001 |
| *p* for interaction: 0.2761 | | | | | |

### **Table S17 Adjusted association of shift work patterns and sleep disruption stratified by self-reported overall health condition**

|  |  | Poor (n= 3956) | | Fair (n= 33827) | | Excellent/good (n= 159622) | |
| --- | --- | --- | --- | --- | --- | --- | --- |
|  |  | OR (95% CI) | p value | OR (95% CI) | p value | OR (95% CI) | p value |
| Shift workers | | |  |  |  |  |  |
|  | non-shift workers | 1.00 (Reference) |  | 1.00 (Reference) |  | 1.00 (Reference) |  |
|  | day shift | 1.39 (1.10, 1.76) | 0.0060 | 1.16 (1.05, 1.27) | 0.0030 | 1.22 (1.16, 1.29) | <0.0001 |
|  | night shift (sometimes/usually) | 1.30 (1.01, 1.67) | 0.0430 | 1.34 (1.21, 1.48) | <0.0001 | 1.38 (1.30, 1.47) | <0.0001 |
|  | night shift (always) | 1.01 (0.67, 1.52) | 0.9670 | 1.44 (1.23, 1.68) | <0.0001 | 1.56 (1.42, 1.72) | <0.0001 |
| *p* for interaction: 0.3808 | | | | | | | |

### **Table S18 Adjusted association of shift work patterns and sleep disruption stratified by hypertension**

|  |  | No (n= 158507) | | Yes (n= 38898) | |
| --- | --- | --- | --- | --- | --- |
|  |  | OR (95% CI) | p value | OR (95% CI) | p value |
| Shift workers | | |  |  |  |
|  | non-shift workers | 1.00 (Reference) |  | 1.00 (Reference) |  |
|  | day shift | 1.22 (1.15, 1.28) | <0.0001 | 1.19 (1.08, 1.31) | <0.0001 |
|  | night shift (sometimes/usually) | 1.39 (1.31, 1.48) | <0.0001 | 1.30 (1.17, 1.45) | <0.0001 |
|  | night shift (always) | 1.50 (1.36, 1.65) | <0.0001 | 1.49 (1.27, 1.76) | <0.0001 |
| *p* for interaction: 0.8545 | | | | | |

### **Table S19 Adjusted association of shift work patterns and sleep disruption stratified by diabetes**

|  |  | No (n= 190949) | | Yes (n= 6456) | |
| --- | --- | --- | --- | --- | --- |
|  |  | OR (95% CI) | p value | OR (95% CI) | p value |
| Shift workers | | |  |  |  |
|  | non-shift workers | 1.00 (Reference) |  | 1.00 (Reference) |  |
|  | day shift | 1.21 (1.16, 1.27) | <0.0001 | 1.18 (0.96, 1.45) | 0.1090 |
|  | night shift (sometimes/usually) | 1.37 (1.30, 1.44) | <0.0001 | 1.36 (1.09, 1.70) | 0.0070 |
|  | night shift (always) | 1.50 (1.38, 1.63) | <0.0001 | 1.53 (1.07, 2.19) | 0.0180 |
| *p* for interaction: 0.9915 | | | | | |

### **Table S20 Adjusted association of shift work patterns and sleep disruption stratified by sleep duration (hours)**

|  |  | <7 (n= 48550) | | 7-9 (n= 147634) | | >9 (n= 1221) | |
| --- | --- | --- | --- | --- | --- | --- | --- |
|  |  | OR (95% CI) | p value | OR (95% CI) | p value | OR (95% CI) | p value |
| Shift workers | | |  |  |  |  |  |
|  | non-shift workers | 1.00 (Reference) |  | 1.00 (Reference) |  | 1.00 (Reference) |  |
|  | day shift | 1.19 (1.10, 1.29) | <0.0001 | 1.22 (1.15, 1.29) | <0.0001 | 1.25 (0.77, 2.01) | 0.3630 |
|  | night shift (sometimes/usually) | 1.31 (1.21, 1.43) | <0.0001 | 1.42 (1.32, 1.51) | <0.0001 | 0.78 (0.45, 1.36) | 0.3830 |
|  | night shift (always) | 1.40 (1.24, 1.59) | <0.0001 | 1.60 (1.44, 1.79) | <0.0001 | 1.20 (0.60, 2.40) | 0.6110 |
| *p* for interaction: 0.1677 | | | | | | | |

### **Table S21 Adjusted association of shift work patterns and sleep disruption stratified by chronotype**

|  |  | Morning chronotype (n=45153) | | More morning (n=64537) | | Middle (n=17640) | | More evening (n=52702) | | Evening chrnotype (n=17373) | |
| --- | --- | --- | --- | --- | --- | --- | --- | --- | --- | --- | --- |
|  |  | OR (95% CI) | *p* value | OR (95% CI) | *p* value | OR (95% CI) | *p* value | OR (95% CI) | *p* value | OR (95% CI) | *p* value |
| Shift workers | | |  |  |  |  |  |  |  |  |  |
|  | non-shift workers | 1.00 (Reference) |  | 1.00 (Reference) |  | 1.00 (Reference) |  | 1.00 (Reference) |  | 1.00 (Reference) |  |
|  | day shift | 1.18 (1.07, 1.29) | 0.0010 | 1.27 (1.17, 1.38) | <0.0001 | 1.24 (1.05, 1.46) | 0.0100 | 1.15 (1.05, 1.26) | 0.0030 | 1.28 (1.10, 1.50) | 0.0010 |
|  | night shift (sometimes/usually) | 1.32 (1.18, 1.47) | <0.0001 | 1.37 (1.24, 1.51) | <0.0001 | 1.49 (1.26, 1.76) | <0.0001 | 1.36 (1.23, 1.50) | <0.0001 | 1.37 (1.17, 1.61) | <0.0001 |
|  | night shift (always) | 1.62 (1.36, 1.93) | <0.0001 | 1.40 (1.18, 1.67) | <0.0001 | 1.35 (1.04, 1.75) | 0.0260 | 1.56 (1.35, 1.82) | <0.0001 | 1.49 (1.22, 1.81) | <0.0001 |
| *p* for interaction: 0.7262 | | | | | | | | | | | |

### **Table S22 Adjusted risk factors** for sleep disruption of participants with different shift work patterns**

|  |  | Non-shift workers (n=167178) | | Day shift workers (n=14928) | | Nightshift(sometimes/usually) (n= 11296) | | Night shift (always) (n=4003) | |
| --- | --- | --- | --- | --- | --- | --- | --- | --- | --- |
|  |  | OR (95% CI) | *p* value | OR (95% CI) | *p* value | OR (95% CI) | *p* value | OR (95% CI) | *p* value |
| **Sociodemographic factors** | |  |  |  |  |  |  |  |  |
| Age group (versus <45) | |  | <0.0001^*^ |  | <0.0001^*^ |  | <0.0001^*^ |  | 0.0800^*^ |
|  | 45-49 | 1.29 (1.22, 1.36) | <0.0001 | 1.12 (0.96, 1.30) | 0.1520 | 1.29 (1.11, 1.50) | 0.0010 | 1.25 (0.99, 1.59) | 0.0590 |
|  | 50-54 | 1.66 (1.57, 1.75) | <0.0001 | 1.41 (1.22, 1.63) | <0.0001 | 1.31 (1.12, 1.53) | 0.0010 | 1.26 (0.99, 1.61) | 0.0610 |
|  | 55-60 | 1.98 (1.87, 2.08) | <0.0001 | 1.45 (1.25, 1.68) | <0.0001 | 1.62 (1.38, 1.91) | <0.0001 | 1.31 (1.01, 1.70) | 0.0450 |
|  | 61-64 | 2.26 (2.13, 2.39) | <0.0001 | 1.75 (1.49, 2.06) | <0.0001 | 1.84 (1.53, 2.21) | <0.0001 | 1.10 (0.81, 1.50) | 0.5340 |
|  | >65 | 2.60 (2.37, 2.84) | <0.0001 | 2.27 (1.72, 2.99) | <0.0001 | 1.68 (1.11, 2.56) | 0.0140 | 0.54 (0.22, 1.35) | 0.1860 |
| Sex (versus female) | |  | 0.1144^*^ |  | 0.0073^*^ |  | 0.1061^*^ |  | 0.2180^*^ |
|  | Male | 1.03 (1.00, 1.06) | 0.0570 | 1.16 (1.05, 1.27) | 0.0020 | 1.10 (0.98, 1.23) | 0.0960 | 0.97 (0.81, 1.17) | 0.7760 |
| Ethnicity (versus white) | |  | <0.0001^*^ |  | <0.0001^*^ |  | <0.0001^*^ |  | 0.0001^*^ |
|  | Mixed race | 1.10 (0.92, 1.31) | 0.2970 | 1.30 (0.86, 1.97) | 0.2130 | 1.19 (0.73, 1.95) | 0.4790 | 0.96 (0.38, 2.41) | 0.9370 |
|  | East Asian | 2.09 (1.70, 2.59) | <0.0001 | 1.50 (0.84, 2.67) | 0.1670 | 1.19 (0.59, 2.41) | 0.6330 | 1.82 (0.73, 4.51) | 0.1960 |
|  | South Asian | 1.39 (1.25, 1.54) | <0.0001 | 1.62 (1.28, 2.05) | <0.0001 | 1.80 (1.40, 2.31) | <0.0001 | 1.74 (1.12, 2.68) | 0.0130 |
|  | Black | 1.58 (1.42, 1.76) | <0.0001 | 1.75 (1.36, 2.25) | <0.0001 | 2.22 (1.81, 2.73) | <0.0001 | 2.05 (1.49, 2.82) | <0.0001 |
|  | Other | 1.46 (1.25, 1.70) | <0.0001 | 1.41 (1.00, 1.98) | 0.0470 | 2.06 (1.54, 2.76) | <0.0001 | 1.85 (1.10, 3.09) | 0.0190 |
| Education (versus Prof Q/NVQ/HND/HNC/Degree or other professional qualification ) | | | 0.0001^*^ |  | 0.8046^*^ |  | 0.8860^*^ |  | 0.3328^*^ |
|  | A levels | 0.90 (0.85, 0.96) | 0.0010 | 1.04 (0.87, 1.25) | 0.6540 | 1.02 (0.82, 1.27) | 0.8820 | 0.79 (0.53, 1.16) | 0.2260 |
|  | O level/GSE/CSE | 0.94 (0.90, 0.98) | 0.0020 | 1.05 (0.95, 1.18) | 0.3440 | 1.03 (0.91, 1.17) | 0.6250 | 0.99 (0.82, 1.20) | 0.8970 |
| Townsend deprivation index (versus quantile 1) | | | <0.0001^*^ |  | 0.9573^*^ |  | 0.1889^*^ |  | 0.0374^*^ |
|  | quantile 2 | 1.07 (1.04, 1.11) | <0.0001 | 1.01 (0.90, 1.13) | 0.8770 | 1.06 (0.93, 1.21) | 0.4060 | 1.20 (0.96, 1.51) | 0.1090 |
|  | quantile 3 | 1.13 (1.08, 1.17) | <0.0001 | 1.00 (0.89, 1.12) | 0.9920 | 1.13 (0.99, 1.29) | 0.0730 | 1.34 (1.07, 1.68) | 0.0100 |
| Household income (versus to less than 18,000 GBP) | | | <0.0001^*^ |  | <0.0001^*^ |  | <0.0001^*^ |  | 0.0015^*^ |
|  | 18,000 to 30,999 | 0.88 (0.83, 0.93) | <0.0001 | 0.80 (0.70, 0.91) | 0.0010 | 0.81 (0.68, 0.96) | 0.0140 | 0.85 (0.66, 1.10) | 0.2150 |
|  | 31,000 to 51,999 | 0.79 (0.74, 0.83) | <0.0001 | 0.65 (0.56, 0.75) | <0.0001 | 0.68 (0.58, 0.81) | <0.0001 | 0.63 (0.48, 0.82) | 0.0010 |
|  | 52,000 to 100,000 | 0.75 (0.71, 0.80) | <0.0001 | 0.60 (0.51, 0.71) | <0.0001 | 0.63 (0.51, 0.76) | <0.0001 | 0.56 (0.40, 0.77) | <0.0001 |
|  | Greater than 100,000 | 0.69 (0.64, 0.75) | <0.0001 | 0.59 (0.45, 0.78) | <0.0001 | 0.51 (0.38, 0.69) | <0.0001 | 0.49 (0.24, 1.00) | 0.0510 |
| Marital status (versus Living with partner ) | |  | <0.0001^*^ |  | 0.0124^*^ |  | 0.7457^*^ |  | 0.1555^*^ |
|  | Not living with partner | 0.91 (0.88, 0.95) | <0.0001 | 0.87 (0.78, 0.97) | 0.0090 | 1.02 (0.91, 1.14) | 0.7650 | 0.87 (0.72, 1.05) | 0.1460 |
| **Anthropometric and lifestyle factors** | |  |  |  |  |  |  |  |  |
| BMI (kg/m2) | |  | <0.0001* |  | 0.0537* |  | 0.0006* |  | 0.0216* |
|  |  | 1.01 (1.01, 1.01) | <0.0001 | 1.01 (1.00, 1.02) | 0.0450 | 1.02 (1.01, 1.03) | <0.0001 | 1.02 (1.00, 1.04) | 0.0210 |
| Smoker (versus never) | |  | 0.0001* |  | 0.7760* |  | 0.0307* |  | 0.0006* |
|  | former | 0.97 (0.94, 1.01) | 0.1010 | 0.97 (0.87, 1.07) | 0.5110 | 1.04 (0.93, 1.16) | 0.5100 | 0.94 (0.78, 1.14) | 0.5530 |
|  | current | 0.89 (0.84, 0.94) | <0.0001 | 0.97 (0.85, 1.12) | 0.7070 | 0.84 (0.72, 0.98) | 0.0250 | 0.64 (0.50, 0.81) | <0.0001 |
| Alcohol (versus daily) | |  | <0.0001* |  | 0.0143* |  | 0.8416* |  | 0.8715* |
|  | 1-4 times/week | 0.97 (0.91, 1.02) | 0.2440 | 1.01 (0.87, 1.18) | 0.9010 | 0.96 (0.80, 1.15) | 0.6650 | 0.93 (0.72, 1.22) | 0.6180 |
|  | Sometimes | 0.93 (0.89, 0.97) | 0.0010 | 0.90 (0.80, 1.02) | 0.1000 | 1.02 (0.88, 1.17) | 0.8210 | 0.98 (0.79, 1.21) | 0.8160 |
|  | Never/special occasion | 0.88 (0.83, 0.92) | <0.0001 | 0.81 (0.69, 0.94) | 0.0060 | 1.05 (0.88, 1.24) | 0.6080 | 0.89 (0.65, 1.22) | 0.4840 |
| Physical activity (versus low) | |  | 0.0836* |  | 0.0073* |  | 0.4614* |  | 0.8487* |
|  | Moderate | 0.99 (0.96, 1.03) | 0.7160 | 0.89 (0.78, 1.01) | 0.0640 | 1.10 (0.94, 1.29) | 0.2250 | 1.07 (0.82, 1.39) | 0.6340 |
|  | High | 0.96 (0.92, 1.00) | 0.0500 | 0.82 (0.72, 0.93) | 0.0020 | 1.05 (0.91, 1.22) | 0.4990 | 1.06 (0.83, 1.37) | 0.6270 |
| Working hours (versus <=40) | |  | <0.0001* |  | 0.0096* |  | 0.9411* |  | 0.2852* |
|  | >40 | 0.90 (0.87, 0.93) | <0.0001 | 0.88 (0.79, 0.98) | 0.0180 | 1.00 (0.90, 1.11) | 0.9840 | 0.93 (0.79, 1.11) | 0.4400 |
| Overall health (versus poor) | |  | <0.0001* |  | <0.0001* |  | <0.0001* |  | 0.0003* |
|  | Fair | 0.62 (0.57, 0.68) | <0.0001 | 0.53 (0.42, 0.67) | <0.0001 | 0.63 (0.49, 0.81) | <0.0001 | 0.90 (0.58, 1.38) | 0.6200 |
|  | Excellent/good | 0.41 (0.38, 0.45) | <0.0001 | 0.38 (0.30, 0.48) | <0.0001 | 0.43 (0.33, 0.55) | <0.0001 | 0.62 (0.41, 0.95) | 0.0270 |
| Hypertension (versus no) | |  | 0.0578* |  | 0.3310* |  | 0.9342* |  | 0.1159* |
|  | Yes | 1.04 (1.00, 1.07) | 0.0630 | 1.05 (0.94, 1.18) | 0.3450 | 0.99 (0.87, 1.12) | 0.8710 | 1.18 (0.97, 1.43) | 0.1070 |
| Diabetes (versus no) | |  | 0.0034* |  | 0.4203* |  | 0.2080* |  | 0.4664* |
|  | Yes | 1.11 (1.03, 1.20) | 0.0040 | 1.09 (0.89, 1.34) | 0.4060 | 1.15 (0.92, 1.44) | 0.2120 | 1.15 (0.80, 1.67) | 0.4420 |
| **Sleep-related characteristics** | |  |  |  |  |  |  |  |  |
| Sleep duration (versus <7h) | |  | <0.0001* |  | <0.0001* |  | <0.0001* |  | 0.0090* |
|  | 7-9 | 1.54 (1.49, 1.59) | <0.0001 | 1.48 (1.34, 1.62) | <0.0001 | 1.37 (1.23, 1.52) | <0.0001 | 1.29 (1.09, 1.52) | 0.0020 |
|  | >9 | 1.46 (1.24, 1.73) | <0.0001 | 1.38 (0.90, 2.13) | 0.1450 | 0.89 (0.55, 1.46) | 0.6440 | 1.07 (0.57, 2.01) | 0.8410 |
| Chronotype (versus middle) | |  | 0.0041* |  | 0.2373* |  | 0.9479* |  | 0.2143* |
|  | morning | 1.11 (1.05, 1.18) | <0.0001 | 1.07 (0.90, 1.27) | 0.4630 | 1.00 (0.83, 1.21) | 0.9610 | 1.31 (0.96, 1.78) | 0.0860 |
|  | morning than evening | 1.08 (1.02, 1.15) | 0.0050 | 1.14 (0.96, 1.36) | 0.1310 | 1.03 (0.86, 1.23) | 0.7610 | 1.15 (0.85, 1.56) | 0.3600 |
|  | evening than morning | 1.08 (1.02, 1.14) | 0.0110 | 1.02 (0.86, 1.22) | 0.7980 | 1.03 (0.85, 1.23) | 0.7860 | 1.30 (0.97, 1.74) | 0.0780 |
|  | evening | 1.12 (1.05, 1.20) | 0.0010 | 1.17 (0.95, 1.45) | 0.1380 | 1.09 (0.87, 1.35) | 0.4600 | 1.36 (0.99, 1.87) | 0.0560 |
| ^*^ The *p* value was obtained using log likelihood ratio test to compare models with and without each covariate. ^**^ The odds ratios and 95% confidence interval for mitigating and exacerbating factors were estimated using adjusted logistic regression models. | | | | | | | | | |

### **Table S23 Adjusted association of shift work patterns and sleep disruption in all participants (without adjustment for sleep duration)**

|  |  | OR (95% CI) | *p* value |
| --- | --- | --- | --- |
| **Sociodemographic factors** | |  |  |
| Age group (versus <45) | |  |  |
|  | 45-49 | 1.27 (1.21, 1.33) | <0.0001 |
|  | 50-54 | 1.60 (1.53, 1.68) | <0.0001 |
|  | 55-60 | 1.91 (1.82, 1.99) | <0.0001 |
|  | 61-64 | 2.16 (2.05, 2.27) | <0.0001 |
|  | >65 | 2.45 (2.25, 2.66) | <0.0001 |
| Sex (versus female) | |  |  |
|  | Male | 1.06 (1.03, 1.09) | <0.0001 |
| Ethnicity (versus white) | |  |  |
|  | Mixed race | 1.16 (1.00, 1.35) | 0.0540 |
|  | East Asian | 1.93 (1.60, 2.33) | <0.0001 |
|  | South Asian | 1.50 (1.38, 1.64) | <0.0001 |
|  | Black | 1.90 (1.75, 2.06) | <0.0001 |
|  | Other | 1.61 (1.42, 1.82) | <0.0001 |
| Education (versus Prof Q/NVQ/HND/HNC/Degree or other professional qualification ) | | | |
|  | A levels | 0.93 (0.88, 0.98) | 0.0050 |
|  | O level/GSE/CSE | 0.97 (0.94, 1.01) | 0.0940 |
| Townsend deprivation index (versus quantile 1) | | |  |
|  | quantile 2 | 1.07 (1.04, 1.10) | <0.0001 |
|  | quantile 3 | 1.13 (1.09, 1.17) | <0.0001 |
| Household income (versus to less than 18,000 GBP) | | | |
|  | 18,000 to 30,999 | 0.86 (0.82, 0.90) | <0.0001 |
|  | 31,000 to 51,999 | 0.75 (0.72, 0.79) | <0.0001 |
|  | 52,000 to 100,000 | 0.72 (0.68, 0.76) | <0.0001 |
|  | Greater than 100,000 | 0.66 (0.61, 0.70) | <0.0001 |
| Marital status (versus Living with partner ) | |  |  |
|  | Not living with partner | 0.93 (0.90, 0.96) | <0.0001 |
| **Anthropometric and lifestyle factors** | |  |  |
| BMI |  | 1.01 (1.01, 1.02) | <0.0001 |
| Smoker (versus never) | |  |  |
|  | former | 0.98 (0.95, 1.01) | 0.1150 |
|  | current | 0.89 (0.85, 0.93) | <0.0001 |
| Alcohol (versus daily) | |  |  |
|  | 1-4 times/week | 0.96 (0.92, 1.01) | 0.1460 |
|  | Sometimes | 0.93 (0.89, 0.96) | <0.0001 |
|  | Never/special occasion | 0.88 (0.84, 0.92) | <0.0001 |
| Physical activity (versus low) | |  |  |
|  | Moderate | 0.99 (0.95, 1.02) | 0.4910 |
|  | High | 0.96 (0.92, 0.99) | 0.0160 |
| Working hours (versus <=40) | |  |  |
|  | >40 | 0.94 (0.91, 0.97) | <0.0001 |
| Overall health (versus poor) | |  |  |
|  | Fair | 0.61 (0.56, 0.65) | <0.0001 |
|  | Excellent/good | 0.39 (0.36, 0.42) | <0.0001 |
| Hypertension | |  |  |
|  | Yes | 1.04 (1.00, 1.07) | 0.0330 |
| Diabetes |  |  |  |
|  | Yes | 1.11 (1.04, 1.18) | 0.0020 |
| **Sleep-related characteristics** | |  |  |
| Chronotype (versus middle) | |  |  |
|  | morning | 1.11 (1.06, 1.17) | <0.0001 |
|  | morning than evening | 1.07 (1.02, 1.13) | 0.0060 |
|  | evening than morning | 1.06 (1.01, 1.12) | 0.0250 |
|  | evening | 1.14 (1.07, 1.21) | <0.0001 |
| **Shift work** | |  |  |
| shiftworkers (versus non-shift workers) | |  |  |
|  | day shift | 1.22 (1.17, 1.28) | <0.0001 |
|  | night shift (sometimes/usually) | 1.40 (1.33, 1.47) | <0.0001 |
|  | night shift (always) | 1.57 (1.45, 1.71) | <0.0001 |
