# Supplementary material for: Associations between shift work patterns and sleep disturbance: an analysis of cross-sectional data from UK Biobank
Source: BMJ Open. 2026 Jan 21;16(1):e102976. doi: 10.1136/bmjopen-2025-102976 (PMC12829381; doi:10.1136/bmjopen-2025-102976)
Supplement: Supplementary file 4 [file bmjopen-2025-102976.reviewer_comments.pdf]

PEER REVIEW HISTORY

BMJ Open publishes all reviews undertaken for accepted manuscripts. Reviewers are asked to complete a checklist review form and are provided with free text boxes to elaborate on their assessment. These free text comments are reproduced below.

ARTICLE DETAILS

Title (Provisional)

Associations between shift work patterns and sleep disturbance: an analysis of cross-sectional data from UK Biobank

Authors

Li, Xianqi; Ray, , David W.; Kyle, Simon D; Smith-Byrne, Karl; Holmes, Leah; Keane, Annie; Parsaeian, Mahboubeh; Travis, Ruth C.; Richmond, Rebecca

---

VERSION 1 - REVIEW

**Reviewer**	**1**
**Name**	**Vitale, Elsa**
**Affiliation**	**Local Health Authority of Bari of the Province of Bari**
**Date**	**30-May-2025**
**COI**	**None**

Many thanks for the opportunity to review this manuscript.

It is well organized and also well written.

Good luck for your paper.

**Reviewer**	**2**
**Name**	**Rudisill, Toni**
**Affiliation**	**West Virginia University, Epidemiology**
**Date**	**06-Aug-2025**
**COI**	**None**

The reviewed paper explores the associations between shift work and the rates of sleep disorders utilizing the UK Biobank data. The study reported important associations between shift work and sleep problems, considering potential covariates identified from previous research. However, there are several minor areas of improvement for this manuscript. I will separate my comments by section.

Abstract:

1. Please specify the data years utilized for this analysis.

Introduction:

1. Pg. 4, line 39-40: overall prevalence of SWSD in the UK or worldwide?

Methodology:

1. There are lots of variables in these models. Many of these could be correlated. It does not appear that collinearity was investigated which could be problematic.

2. The authors did not specify their what software they used for data management and analyses. They also did not specify their alpha for significance testing.

3. Page 8, line 46-47: The word modified should be modify.

4. How much missing data were there? This isn't specified.

5. it isn't clear what data years were used for this specific analysis. It is only mentioned

that the UK Biobank recruited individuals between 2006-2010. If the data were from that time period only, this study is very outdated.

6. In the Study Design and Participants section, the authors describe the UK Biobank. However, does the UK Biobank only include adults? I feel the authors need to add what were their specific inclusion or exclusion criteria here as well in addition to the description of the dataset. This would help clarify who they specifically included/analyzed in this study.

Results:

1. Please elaborate on 'materially deprived'. That is vague (line52, Page 9).

2. Page 10, line 11: One should start a sentence with words not numbers.

3. Circadian rhythm disruption can exacerbate cancer risks – is this something that was covered in the Biobank survey? If so, it would be of interest to report these as well.

VERSION 1 - AUTHOR RESPONSE

Reviewer: 1
Dr. Elsa Vitale, Local Health Authority of Bari of the Province of Bari
Comments to the Author:
Many thanks for the opportunity to review this manuscript.

It is well organized and also well written.
Good luck for your paper.

We thank the reviewer for their positive comments on the manuscript.

Reviewer: 2
Dr. Toni Rudisill, West Virginia University
Comments to the Author:
The reviewed paper explores the associations between shift work and the rates of sleep disorders utilizing the UK Biobank data. The study reported important associations between shift work and sleep problems, considering potential covariates identified from previous research. However, there are several minor areas of improvement for this manuscript. I will separate my comments by section.

We thank the reviewer for their positive feedback on the manuscript and have carefully addressed their additional comments to improve the quality of the paper. References refer to the marked up version of the manuscript.

Abstract:
1. Please specify the data years utilized for this analysis.

In the "Participants" section of the abstract, we state the sample comprises UK Biobank participants at baseline (2006 – 2010).

Introduction:
1. Pg. 4, line 39-40: overall prevalence of SWSD in the UK or worldwide?

In the Introduction (**page 3, lines 17-19),** we state that a systematic review and meta-analysis reported that the overall prevalence of SWSD was 26.5%. This comprised individual studies conducted in 16 countries worldwide, including both low and-middle income and high-income settings. We have added this information to the Introduction.

Methodology:
1. There are lots of variables in these models. Many of these could be correlated. It does not appear that collinearity was investigated which could be problematic.

Thank you for raising this important point. We assessed potential multicollinearity among covariates using Variance Inflation Factors (VIFs) derived from ordinary least squares regression including the same set of predictors. Across all models, mean VIF values were below 2, and no variable exceeded the commonly used threshold of 10, indicating no concerning multicollinearity. The only variable with slightly elevated VIF values was self-reported overall health when entered as a categorical variable, which reflects the creation of multiple dummy variables rather than correlation with other predictors. As multicollinearity primarily affects standard errors rather than coefficient estimates, and given that this effect arises from variable coding, we do not believe it materially influences our results. For transparency of reporting, we have included the VIF values for the variables entered into the Model 3 as a supplementary table (**Table S3**). We now state in the Statistical Analysis part of the Methods that "Multicollinearity of the variables included in the models was assessed using Variance Inflation Factors (VIFs) derived from ordinary least squares regression including the same set of predictors." (**page 7, lines 8-10**) and in the Results that, "There was limited evidence for multicollinearity among

covariates included in the models, with a mean VIF value below 2 for the most adjusted model (Model 3) (Table S3)." (**page 9, line 1-2**).

2. The authors did not specify their what software they used for data management and analyses. They also did not specify their alpha for significance testing.

In the Statistical Analysis section (**page 7, lines 19-20**) we state that, "All data management and analyses were conducted with Stata version 18 (StataCorp LLC, College Station, TX, USA)." Thank you for also noting the omission of the alpha for significance testing. We have now specified our α level in the Statistical Analysis section (**page 7, line 20**: "All hypothesis tests were two-tailed with $\alpha = 0.05$." We also acknowledge, consistent with Sterne and Davey Smith's *"Sifting the evidence—what's wrong with significance tests?"* (BMJ, 2001), that strict reliance on arbitrary significance thresholds can be misleading. Accordingly, in the manuscript we emphasize interpretation of effect sizes and confidence intervals rather than dichotomous "significant/non-significant" labels.

3. Page 8, line 46-47: The word modified should be modify.

Thank you for highlighting this typo which has now been corrected.

4. How much missing data were there? This isn't specified.

Thank you for raising this. The proportion of missing data for all participant characteristics considered in the study are now presented in **Table S1**. We have also added more detail on the total sample sizes for each of the three main models considered in **Table 1**. In the Statistical Analysis section of the Methods, we now state "The amount of missingness for each variable is highlighted in Table S1, and for each model in Table 1." (page 7, lines 7-8).

5. It isn't clear what data years were used for this specific analysis. It is only mentioned that the UK Biobank recruited individuals between 2006-2010. If the data were from that time period only, this study is very outdated.

Thank you for the comment. While our analysis uses baseline data collected between 2006–2010, these data remain highly relevant for investigating associations between shift work and sleep disruption. The UK Biobank's large, well-characterized cohort provides substantial variability in both shift work exposure and sleep measures, allowing for robust analysis. Although recruitment occurred over a decade ago, the biological and behavioral relationships underlying the relationship between shift work and sleep disruption are unlikely to have changed substantially since then. While follow-up data on shift work have been collected for a subset of participants, the proportion is limited, and given that participants were aged 40-69 at baseline, the number of people engaged in shift work has diminished considerably over time as more participants have retired or reduced their working time (n=7,693 in the most recent assessment in 2023 vs n=48,975 at baseline). This therefore makes baseline data the most appropriate source for examining these associations between shift work and sleep disruption in this study. Nonetheless, in the limitations section of the Discussion we now state, "Furthermore, since our analysis used data collected between 2006-2010, an assessment of the associations between shift work and sleep disruption in more contemporaneous cohorts is warranted." (**page 12, lines 20-22**).

6. In the Study Design and Participants section, the authors describe the UK Biobank. However, does the UK Biobank only include adults? I feel the authors need to add what were their specific

inclusion or exclusion criteria here as well in addition to the description of the dataset. This would help clarify who they specifically included/analyzed in this study.

We apologise for not specifying the age range and inclusion criteria of the UK Biobank participants. The study recruited adults aged 40-69 years who were registered with the UK National Health Service (NHS). We have now provided details of this in the "Study Design and Participants" section as well as a description of the recruitment process, which involved sending invitations to about 9 million people who were registered with the UK National Health Service (NHS), of whom 5.5% (~500,000) responded positively and attended an assessment centre (**page 4, lines 20-22**). We also direct the reader to additional references where more details of the UK Biobank study are available.(Cox, 2018; Sudlow et al., 2015)

Results:
1. Please elaborate on 'materially deprived'. That is vague (line52, Page 9).

We have now clarified this description, stating now that shift workers tended "to have a higher degree of material deprivation (as measured using the Townsend deprivation index)" (**page 8, lines 11-12**).

2. Page 10, line 11: One should start a sentence with words not numbers.

Rather than writing out a five-digit number, we have restructured the sentence to read "Among non-shift workers, 33,012 (14.0%) were classified as having sleep disturbance, compared with 9,469 (19.3%) among shift workers" (**page 8, lines 21-22**).

3. Circadian rhythm disruption can exacerbate cancer risks – is this something that was covered in the Biobank survey? If so, it would be of interest to report these as well.

While circadian rhythm disruption and its potential link to cancer risk is indeed an important area of research, this is beyond the scope of the present study, which is focused specifically on examining the association between shift work and sleep disturbance within the UK Biobank cohort. Data on cancer incidence or circadian-related biomarkers were not included in our analytical framework and would be more appropriately addressed in future research.

References

Cox, N. (2018). UK Biobank shares the promise of big data. *Nature, 562*(7726), 194-195. doi:10.1038/d41586-018-06948-3

Sudlow, C., Gallacher, J., Allen, N., Beral, V., Burton, P., Danesh, J., . . . Collins, R. (2015). UK biobank: an open access resource for identifying the causes of a wide range of complex diseases of middle and old age. *PLoS Med, 12*(3), e1001779. doi:10.1371/journal.pmed.1001779

VERSION 2 - REVIEW

**Reviewer**	**2**
**Name**	**Rudisill, Toni**
**Affiliation**	**West Virginia University, Epidemiology**

Date **25-Nov-2025**

COI

The study authors have addressed all my comments from the previous version. This version is much improved than the last.
